# DNA barcoding and TLC as tools to properly identify natural populations of the Mexican medicinal species *Galphimia glauca* Cav

**DOI:** 10.1371/journal.pone.0217313

**Published:** 2019-05-28

**Authors:** Reinier Gesto-Borroto, Alexandre Cardoso-Taketa, Jessica P. Yactayo-Chang, Karina Medina-Jiménez, Claudia Hornung-Leoni, Argelia Lorence, Maria Luisa Villarreal

**Affiliations:** 1 Doctorado en Ciencias, Centro de Investigación en Dinámica Celular, Instituto de Investigación en Ciencias Básicas y Aplicadas, Universidad Autónoma del Estado de Morelos, Morelos, México; 2 Laboratorio de Investigación en Plantas Medicinales, Centro de Investigación en Biotecnología, Universidad Autónoma del Estado de Morelos, Morelos, México; 3 Arkansas Biosciences Institute, Arkansas State University, Jonesboro, Arkansas, United States of America; 4 Centro de Investigaciones Biológicas, Instituto de Ciencias Básicas e Ingeniería Universidad Autónoma del Estado de Hidalgo, Hidalgo, México; Institute for Biological Research, SERBIA

## Abstract

*Galphimia glauca* is a plant that is endemic to Mexico and has been commonly used since pre-Hispanic times to treat various illnesses, including central nervous system disorders and inflammation. The first studies investigating a natural population of *G*. *glauca* in Mexico showed that the plant has anxiolytic and sedative activities in mice and humans. The plant’s bioactive compounds were isolated and identified, and they belong to a family of *nor*-secofriedelanes called galphimines. The integration of DNA barcoding and thin-layer chromatography analysis was performed to clarify whether the botanical classification of the populations in the study, which were collected in different regions of Mexico, as *G*. *glauca* was correct or if the populations consist of more than one species of the genus *Galphimia*. We employed six DNA barcodes (*matK*, *rbcL*, *rpoC1*, *psbA-trnH*, *ITS1* and *ITS2*) that were analyzed individually and in combination and then compared each other, to indicate differences among the studied populations. In the phylogenetic analysis, *ITS1* and *ITS2* markers as well as the combination of all DNA regions were the most efficient for discriminating the population studied. The thin-layer chromatography analysis exhibited four principal chemical profiles, one of which corresponded to the populations that produced galphimines. DNA barcoding was consistent and enabled us to differentiate the populations that produce galphimines from those that do not. The results of this investigation suggest that the studied populations belong to at least four different species of the genus *Galphimia*. The phylogenetic analysis and the thin-layer chromatography chemical profiles were convenient tools for establishing a strong relationship between the genotype and phenotype of the studied populations and could be used for quality control purposes to prepare herbal medicines from plants of the genus *Galphimia*.

## Introduction

*Galphimia glauca* Cav. (Malpighiaceae), is a plant that is endemic to Mexico and has been traditionally used to treat different ailments, including central nervous system disorders and inflammation [1]. *G*. *glauca* is widely distributed in Mexico [2]; however, scientific investigations of the phytochemical and pharmacological properties of this plant have been limited to populations growing in specific localities of the country. The first studies were conducted in natural populations growing in Doctor Mora, Guanajuato, and showed that the plant has anxiolytic and sedative activities in both mice [3] and humans [4]. The bioactive compounds were isolated and identified, and they correspond to a family of *nor*-secofriedelanes known as galphimines [5,6]. Subsequently, two metabolomic analyses were carried out in seven populations of the plant collected in the states of Chiapas, Jalisco, Morelos and Querétaro. The results of these investigations showed that only two populations produce galphimines [3,7] exhibiting anxiolytic and sedative activities in mice; however, all of them had anti-inflammatory activity in mice [7]. These results indicate that although these plants are morphologically similar, they are different in respect to their biological activity and metabolic profile; these differences may be observed due to divergent environmental conditions or because the plants consist of different botanical species.

An actual procedure to identify species of organisms is DNA barcoding, which includes a short segment of DNA from a standard and agreed-upon position in the genome of the nucleus and organelles [8]. Since the introduction of this method, DNA barcoding has been widely used in ecological, environmental and conservation studies [9–12] or forensic genetics [13,14], as well for the authentication of medicinal plants of different families [15–24]. From the Malpighiaceae family using molecular markers, only a small number of studies of phylogenetic analysis have been published [25–29].

In a previous study conducted by our research group, a DNA barcoding analysis was carried out in seven populations botanically classified as *G*. *glauca* by the HUMO Herbarium, CIByC (Centro de Investigación en Biodiversidad y Conservación), UAEM, Mexico. In that work, three genes, *matK*, *rbcL*, and *rpoC1*, were used for the molecular analysis, and the results suggested that the seven populations in the study may have belonged to at least three different species of the genus *Galphimia* [30].

In the present investigation, individuals from eight natural populations were collected and botanically classified as *G*. *glauca* by the HUMO Herbarium, CIByC UAEM, Mexico and the HGOM Herbarium, CIB (Centro de Investigaciones Biológicas), UAEH, Mexico. These specimens (six per population) were collected in different geographical locations in Mexico. To determine if all of the populations were correctly classified as *G*. *glauca* or if they belong to different species of the genus *Galphimia*, they were studied using six DNA barcode genes (*matK*, *rbcL*, and *rpoC1*, used in our first study; and *psbA-trnH*, *ITS1* and *ITS2*, employed in this study for the first time). In addition, metabolic profiling was performed using thin-layer chromatography.

## Materials and methods

### Plant material

Plant material (six individuals per population) was collected in the summer of 2015 and 2016 (August and September) from eight different populations growing in five states of Mexico (Table 1): Doctor Mora, Guanajuato (GM); Jalpan de Serra (QJ) and Cadereyta (QC), Querétaro; Zimapán, Hidalgo (HZ); Cuernavaca (MC), Miacatlán (MM) and Santa Catarina (MS), Morelos; and Ciudad Valles, San Luis Potosí (SV). Specimens were deposited at the HUMO Herbarium, CIByC (Centro de Investigación en Biodiversidad y Conservación), UAEM, and at the HGOM Herbarium, CIB (Centro de Investigaciones Biológicas), UAEH, in Mexico. Leaves of *G*. *glauca* were kept at −80°C for DNA extraction. Similar populations from GM, QJ, MC and MM were studied in our previous investigation conducted six years prior [30].

**Table 1:** General data for the studied *Galphimia glauca* populations.

Population	Voucher (No.)	Locality	Date and time of collection	Position	Altitude (m)	Ecosystem
GM	15189	Doctor Mora, Guanajuato	August 14, 2015, 11:00–12:00 h	N 21.0874 W 100.1922	2120	Semidry secondary vegetation
QC	35894	Cadereyta, Querétaro	September 10, 2015, 9:00–10:00 h	N 20.6991 W 99.7250	2030	Semidry secondary vegetation
QJ	15018	Jalpan de Serra, Querétaro	September 10, 2015, 16:00–17:00 h	N 21.2850 W 99.2857	1548	Pine-oak forest
MC	15011	Cuernavaca, Morelos	August 24, 2015, 10:00–11:00 h	N 18.5891 W 99.1348	2204	Pine-oak forest
MM	15426	Miacatlán, Morelos	August 31, 2015, 11:00–12:00 h	N 18.45.57 W 99.2175	1004	Seasonal-dry tropical forest secondary vegetation
MS	35896	Santa Catarina, Morelos	August 24, 2015, 12:00–13:00 h	N 18.9756 W 99.1709	1600	Seasonal-dry tropical forest secondary vegetation
HZ	1483	Zimapán, Hidalgo	August 20, 2016, 12:00–13:00 h	N 20.9696 W 99.3138	1999	Semidry secondary vegetation
SV	35895	Ciudad Valles, San Luis Potosí	September 17, 2016, 10:00–11:00 h	N 22.2225 W 98.97.1	310	Seasonal-dry tropical forest secondary vegetation

### DNA extraction

Genomic DNA was isolated from frozen leaves (10 mg) using the “Puregene DNA Purification” kit (Qiagen) following the manufacturer’s protocol. The DNA samples were dissolved (50 ng/μL) in T_10_E_1_ buffer and kept at −20°C before being used for PCR amplification. The quality and quantity of DNA were determined in a NanoDrop^TM^ 8000 spectrophotometer measuring the absorbance at 260 and 280 nm and by visual inspection after being run on an agarose gel.

### Oligonucleotides

The oligonucleotides used for PCR amplification of *matK*, *rbcL* and *rpoC1* genes were previously reported [28,30] (Table 2). The oligonucleotides for the *ITS* (including *ITS1* and *ITS2* genes) [18,31–36] and *psbA-trnH* [31,37–42] regions were selected by an exhaustive search of the previously reported studies investigating angiosperm plants using these markers and by using the BLASTn routine in GenBank database (http://www.ncbi.nlm.nih.gov/genbank/) (Table 2).

**Table 2:** Oligonucleotide sequences for DNA barcodes.

Gene	Sequence forward	Sequence reverse
*matK*	GAG GGG TTT GCA GTC ATT GT	CCA ATG ACC CAA TCA AAG GA
*rbcL*	GAA GGG TCT GTT ACT AAC ATG	TCC CCT TCA AGT TTA CCT AC
*rpoC1*	GTG GAT ACA CTT CTT GAT AAT GG	CCA TAA GCA TAT CTT GAG TTG G
*ITS*	GTC CAC TGA ACC TTA TCA TTT AG	TCC TCC GCT TAT TGA TAT GC
*psbA-trnH*	GTT ATG CAT GAA CGT AA TGC TC	CGC GCA TGG TGG ATT CAC AAT CC

### PCR amplification, purification and sequencing

For all six genes, PCR amplification was performed in a Robocycler Gradient 96 (Stratagene) using the following program: a first step of 4 min at 94°C followed by 35 cycles of 1 min at 94°C, 1 min at 53°C, and 1 min at 72°C. A final extension of 10 min at 72°C was performed at the end. PCR analyses were performed in a final volume of 50 μL using 25 μL of Go Taq Green Master Mix, 19 μL of nuclease-free water, 2 μL of each oligonucleotide, and 2 μL of DNA sample. The PCR products were observed in an agarose gel (1%) to verify the presence of unique amplicons. The PCR products were purified using “QIAquick PCR Purification” (Qiagen) following the manufacturer’s instructions. To quantify the PCR product, 2 μL was used in a NanoDrop^TM^ 8000 spectrophotometer, which measured the absorbance at 260 and 280 nm. The final concentration of the amplicons was adjusted to 50 ng/μL before the sequencing process.

All PCR products were sequenced in both directions. For sequencing, 1.4 μL of DNA and 1.2 μL of each primer were added to 1 μL of BigDye 3.1 reaction mixture (Applied BioSystems). To complete the reaction volume to 6.4 μL, 1 μL of sequencing buffer was added. The reaction cycle was performed at 96°C for 10 sec, 50°C for 5 sec and 60°C for 10 min. After the reaction cycle, water was added to each tube to complete the volume to ~25 μL. The reactions were purified in Sephadex G50 superfine 96-well filter plates. The collected samples were run on an Applied Biosystems 3730XL capillary instrument (DNA Sequencing Facility, University of Chicago Cancer Research Center). All sequences were visually inspected and edited using Chromas 2.6.5 (Technelysium Pty Ltd). The six forward and six reverse sequences were used to obtain a consensus sequence for each marker in each population. All consensus sequences were deposited in GenBank, and accession numbers were obtained (Table 3).

**Table 3:** Accession number of the consensus sequences deposited in GenBank.

Population	*matK*	*rbcL*	*rbcL*	*ITS*	*psbA-trnH*
GM				MH842122	MK805053
HZ	MK033195	MK033203	MK033199	MH842123	MK805054
MC				MH842124	MK805055
MM				MH842125	MK805056
MS	MK033196	MK033205	MK033201	MH842126	MK805057
QC	MK033197	MK033204	MK033200	MH842127	MK805058
QJ				MH842128	MK805059
SV	MK033198	MK033206	MK033202	MH842129	MK805060

### DNA barcoding analysis

#### Distance-based method

For all six DNA barcodes, the genetic variability using the DnaSP6 program [43] was determined. The alignment and sequence average lengths, the number of sites with gaps, the number of polymorphic and parsimony informative sites, the nucleotide diversity and the number of haplotypes were considered for this analysis. Moreover, the intraspecific and interspecific distances were obtained using Kimura’s 2-parameter (K2P) nucleotide evolution model [44] in the MEGA 7 program [45].

#### Phylogenetic analysis

For the phylogenetic analysis, we used all the available sequences in the GenBank database (http://www.ncbi.nlm.nih.gov/genbank/) for the genus *Galphimia*, including all six markers in study, as well as sequences reported previously for *G*. *glauca* [30]. Sequences from other phylogenetically closely related species and genera, as well as not closely related genera, were also selected for phylogenetic out groups (Table 4).

**Table 4:** Accession number of sequences used in the phylogenetic study.

Gene	Accession number	Species
*matK*	JX088054	*Galphimia glauca* Cav.
	JX088053	*Galphimia glauca* Cav.
	JX088058	*Galphimia glauca* Cav.
	JX088055	*Galphimia glauca* Cav.
	AB233800	*Galphimia glauca* Cav.
	HQ247275	*Galphimia brasiliensis* (L.) A.Juss.
	MF349853	*Galphimia gracilis* Bartl.
	HQ247276	*Galphimia mirandae* C.E.Anderson
	HQ247277	*Galphimia speciosa* C.E.Anderson
	JQ588187	*Byrsonima crassifolia* (L.) Kunth
	KU556672	*Silybum marianum* (L.) Gaertn.
	KX677063	*Vaccinium oxycoccos* L.
*rbcL*	JX125620	*Galphimia glauca* Cav.
	JX125622	*Galphimia glauca* Cav.
	JX125624	*Galphimia glauca* Cav.
	JX125621	*Galphimia glauca* Cav.
	AB233904	*Galphimia glauca* Cav.
	KM197480	*Galphimia australis* Chodat
	HQ247487	*Galphimia brasiliensis* (L.) A.Juss.
	AF344475	*Galphimia gracilis* Bartl.
	HQ247488	*Galphimia multicaulis* A.Juss.
	HQ247489	*Galphimia speciosa* C.E.Anderson
	JX664036	*Byrsonima crassifolia* (L.) Kunth
*rpoC1*	JX125630	*Galphimia glauca* Cav.
	JX125628	*Galphimia glauca* Cav.
	JX125625	*Galphimia glauca* Cav.
	JX125627	*Galphimia glauca* Cav
	FJ038768	*Bunchosia* sp. MAG-2009
	GQ429092	*Byrsonima crassifolia* (L.) Kunth
	KC737454	*Mytilaria laosensis* Lecomte
	KC481618	*Oxalis acetosella* L.
*ITS1* and *ITS2*	KR087554	*Galphimia australis* Chodat
	MF349119	*Galphimia gracilis* Bartl.
	AY137299	*Acridocarpus macrocalyx* Engl.
	AF436780	*Aspidopterys elliptica* A.Juss.
	KR087517	*Byrsonima crassifolia* (L.) Kunth
	KP675783	*Melia azedarach* L.
	AJ012364	*Nicotiana tabacum* L.
*psbA-trnH*	GQ982369.1	*Spachea membranacea* Cuatrec.
	KY027037.1	*Byrsonima coccolobifolia* Kunth
	MH826615.1	*Elaeocarpus amplifolius* Schltr.
	AB492621.1	*Mitella furusei* Ohwi

The sequences were aligned using Clustal W [46] with default parameters, and the phylogenetic analysis was performed in MEGA 7.0 [45]. For each marker, we generated separate Neighbor-joining (NJ) as well as Maximum Likelihood (ML), and Maximum Parsimony (MP) phylogenetic trees (S1 Appendix) based on the K2P nucleotide evolution model [44], data-specific model [47] and close-neighbor-interchange algorithm [47], respectively. The sequences for all six markers were combined with a total length of 2964–3022 bp, and phylogenetic analysis was performed using the NJ method. Node support was assessed via 1000 bootstrap replicates. The resulting phylogenetic trees were edited using the iTOL program (http://itol.embl.de/) [48]. We employed the NJ, ML, and MP methods because they have superior computationally intensive analysis in comparison to other methods and to confirm that the choice of phylogenetic method algorithms did not change DNA barcode results.

### Thin-layer chromatography

Methanolic extracts were prepared for all eight populations (six individual extracts for each population). The samples (leaves) were dried for 3–4 days in a cool and dry place without direct sunlight. Samples were then pulverized by mortar and pestle. Pulverized dried material (100 mg) for each sample was mixed with 1 mL MeOH. Samples were vortexed for 2 min, sonicated for 15 min and then centrifuged at 10,000 rpm for 15 min. The supernatant was removed, and the material residue containing the pellet was reprocessed four times to achieve exhaustive extraction. All supernatants were combined and applied directly in thin-layer chromatography (TLC) plates, which were eluted using a mobile phase of CHCl_3_:CH_3_COOCH_2_CH_3_ (2:1 v/v). TLC plates (silica gel 60 UV_254_ precoated, 10 × 10 cm, 200 μm layer thickness, aluminum-backed, mean particle size of 10–12 μm, particle size distribution of 5–20 μm) were stained with a spray solution containing vanillin/H_2_SO_4_ (1 g of vanillin in 100 mL of H_2_SO_4_) and heated at 120°C until maximum color formation.

## Results

Eight populations botanically classified as *G*. *glauca* (six individuals per population) were analyzed using a molecular approach by DNA barcodes and phytochemical analysis by TLC. Individuals from four of these populations (GM, MC, MM and QJ) were studied previously by our group [30]. In this new research, we considered these four populations because three new DNA barcodes (*psbA-trnH*, *ITS1* and *ITS2*) were introduced in the molecular analysis and to compare the actual chemical profiles by TLC analysis of individuals collected in the four abovementioned localities six years previously.

### DNA barcoding analysis

#### DNA barcode features and distance-based methods

In this study, we used six different molecular markers (*matK*, *rbcL*, *rpoC1*, *psbA-trnH*, *ITS1* and *ITS2*) that have been previously reported with high levels of species identification in angiosperm [34,49–51]. For each marker, all six individuals in each population showed 100% identical DNA sequences, with the exception of *psbA-trnH*, which showed between 2 and 4 nonspecific nucleotides in the sequences. The sequences for *matK*, *rbcL* and *rpoC1* that were obtained in our previous work [30] were included in this work to make an accurate analysis and comparison of the genetic variability of the *G*. *glauca* populations in study. The lengths of the DNA fragments that were generated were 793–829, 669, 599, 433–449, 256–258 and 214–218 bp for *matK*, *rbcL*, *rpoC1*, *psbA-trnH*, *ITS1* and *ITS2*, respectively. The sequences were similar among members of each population, but single nucleotide polymorphisms were observed among all six markers.

The largest sequences of all DNA barcodes were obtained for *matK*, with median and alignment lengths of 797.5 and 829 bp, respectively. The shortest sequences were recorded for the *ITS2* marker, with median and alignment lengths of 216.8 and 221 bp, respectively. For the *rbcL* barcode, smaller values of variability were obtained without any parsimony informative site and a nucleotide diversity of 0.003. In the same way, *matK* and *rpoC1* showed low genetic variability with only 2 parsimony informative sites and a nucleotide diversity of 0.004 and 0.003, respectively (Table 5). The intergenic regions *psbA-trnH*, *ITS1* and *ITS2* were the most variable DNA barcodes in this study. In particular, the *psbA-trnH* intergenic spacer showed the highest values of polymorphic (equal to 48) and parsimony-informative (equal to 18) sites. The spacer regions *ITS2* exhibited the highest value of nucleotide diversity (equal to 0.055). Conversely, *psbA-trnH*, *ITS1* and *ITS2* markers presented the largest number of haplotypes (equal to 4) (Table 5).

**Table 5:** Molecular features of *Galphimia* populations DNA barcodes.

Barcode	Average sequence length (bp)	Sequence alignment length (bp)	No. of sites with gaps	No. of polymorphic sites	No. of parsimony informative sites	Nucleotide diversity	No. of haplotypes
*matK*	797.5	829	36	11	2	0.004	3
*rbcL*	669	669	0	9	0	0.003	3
*rpoC1*	599	599	0	5	2	0.003	3
*ITS1*	257.8	261	8	34	10	0.044	4
*ITS2*	216.8	221	10	40	7	0.055	4
*psbA-trnH*	443	475	58	48	18	0.042	4

**Fig 1:**
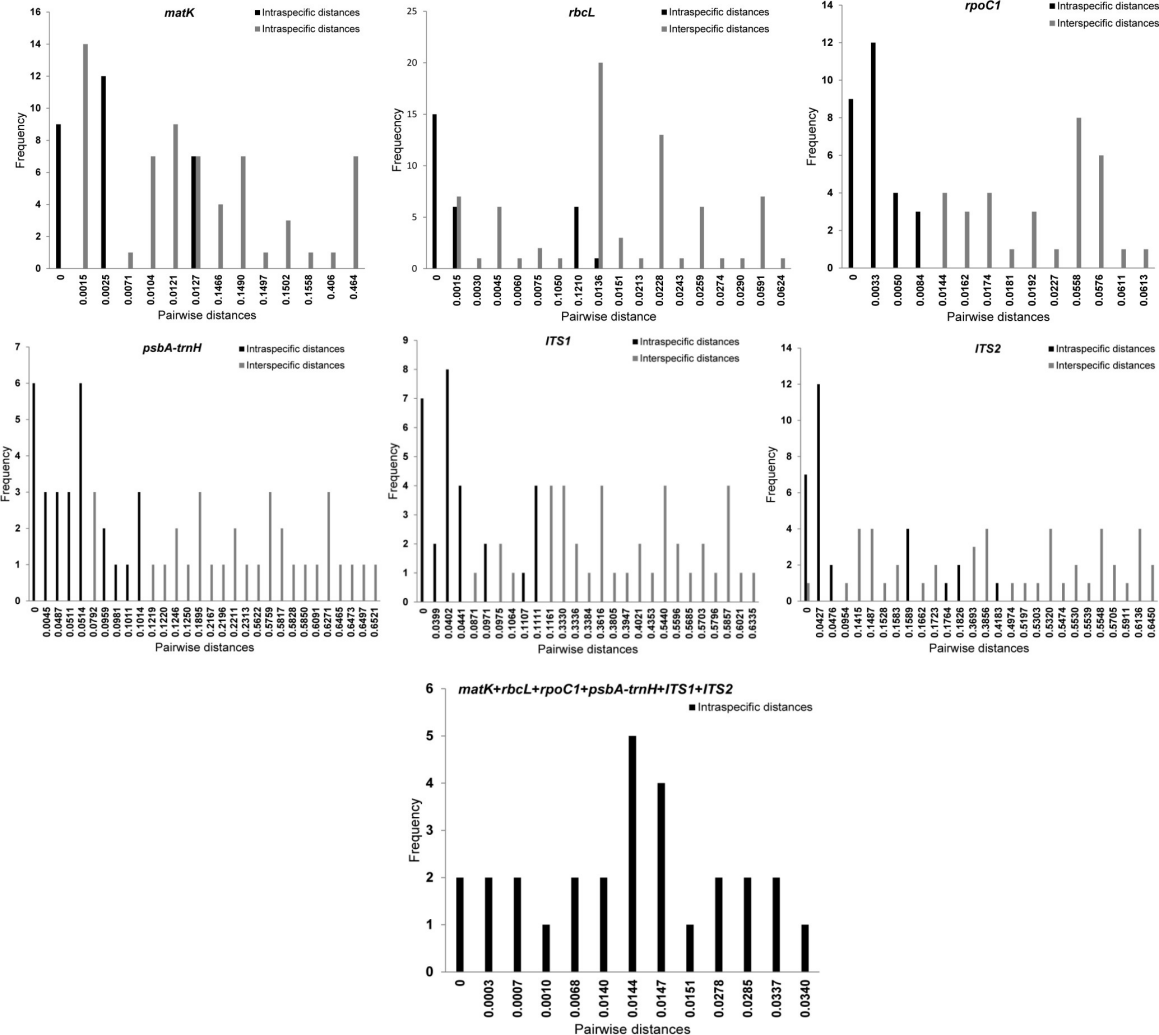
Frequency histograms of the pairwise intra- and interspecific distances for barcodes *matK*, *rbcL*, *rpoC1*, *psbA-trnH*, *ITS1* and *ITS2* and for all combined barcodes obtained for *Galphimia* populations.

The intraspecific distances were evaluated considering the botanical classification as *G*. *glauca* of all populations in study. For the interspecific distances, the comparison was performed between the populations of *G*. *glauca* in the study and the species selected as out groups for the analysis (Fig 1). In the case of the intraspecific distances, *matK*, *rbcL* and *rpoC1* showed low values, that is, below 0.0127. However, *psbA-trnH*, *ITS1* and *ITS2* exhibited high values of intraspecific distances, ranging from 0.0616–0.172, 0.0399–0.111 and 0.0427–0.1826, respectively (Fig 1).

#### Phylogenetic analysis

**Fig 2:**
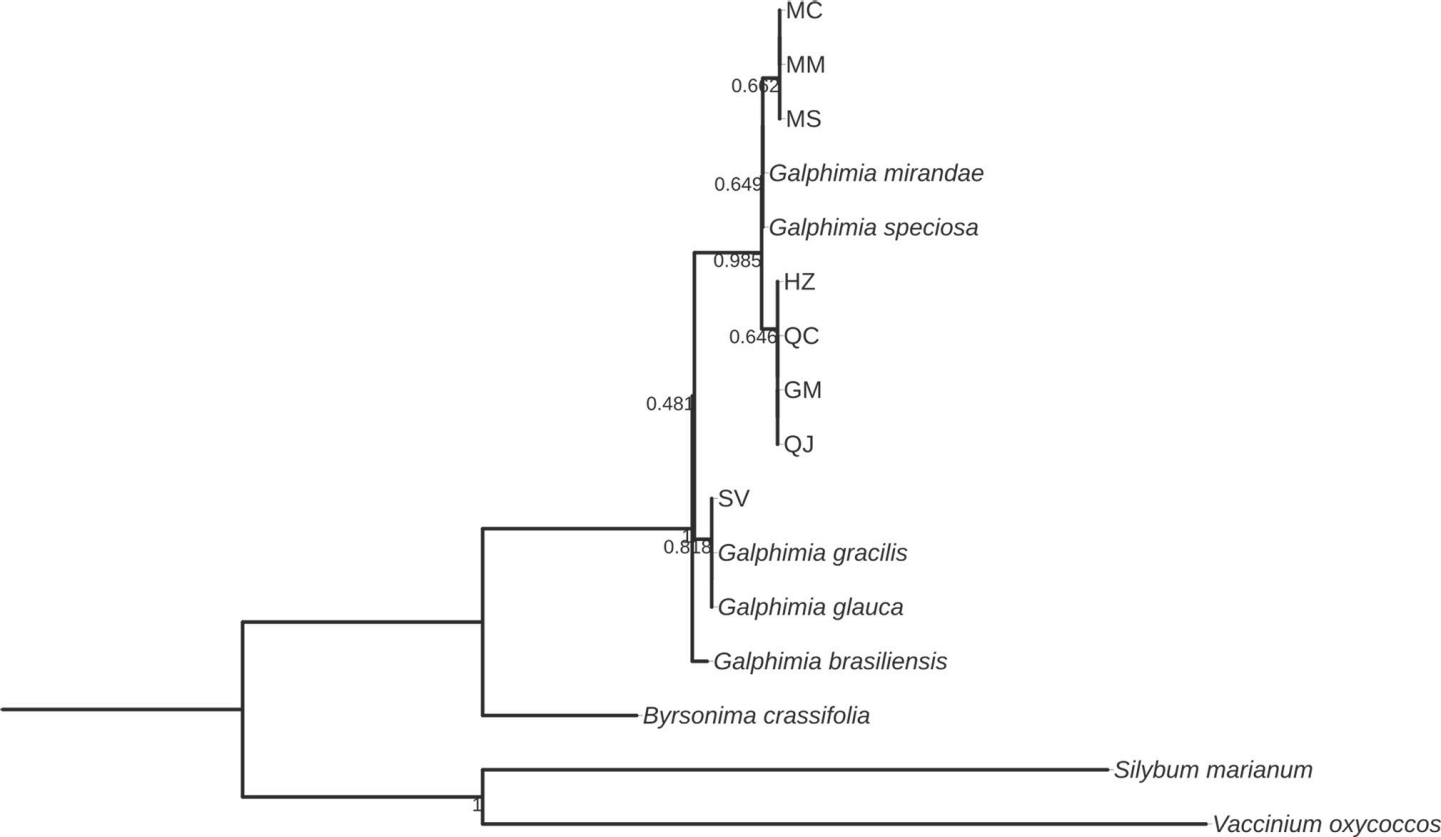
Bootstrap consensus tree generated by the Neighbor-joining method for *matK* sequences obtained for *Galphimia* populations. Numbers below the branches are bootstrap values expressed as a percentage of 1000 replicates.

**Fig 3:**
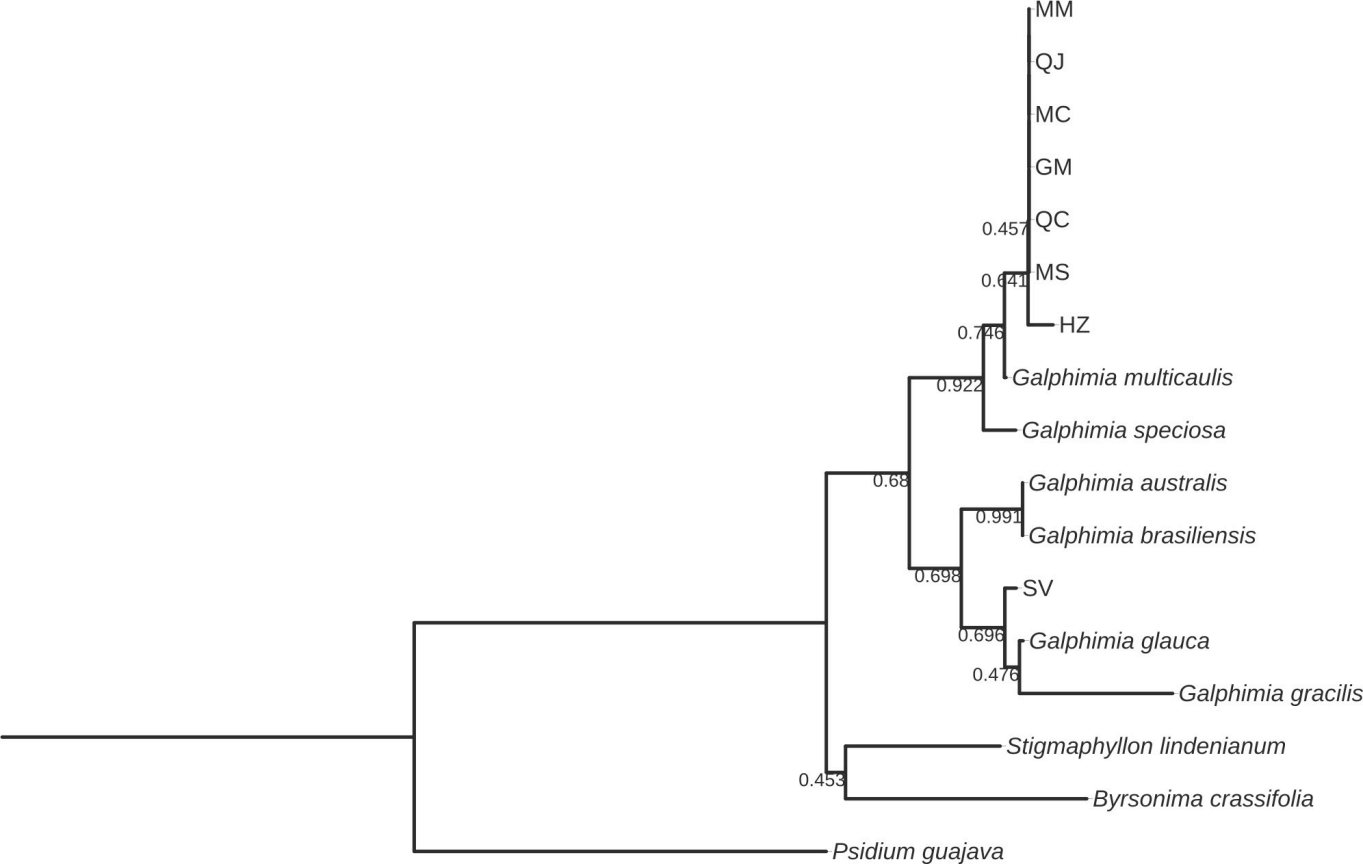
Bootstrap consensus tree generated by the Neighbor-joining method for *rbcL* sequences obtained for *Galphimia* populations. Numbers below the branches are bootstrap values expressed as a percentage of 1000 replicates.

**Fig 4:**
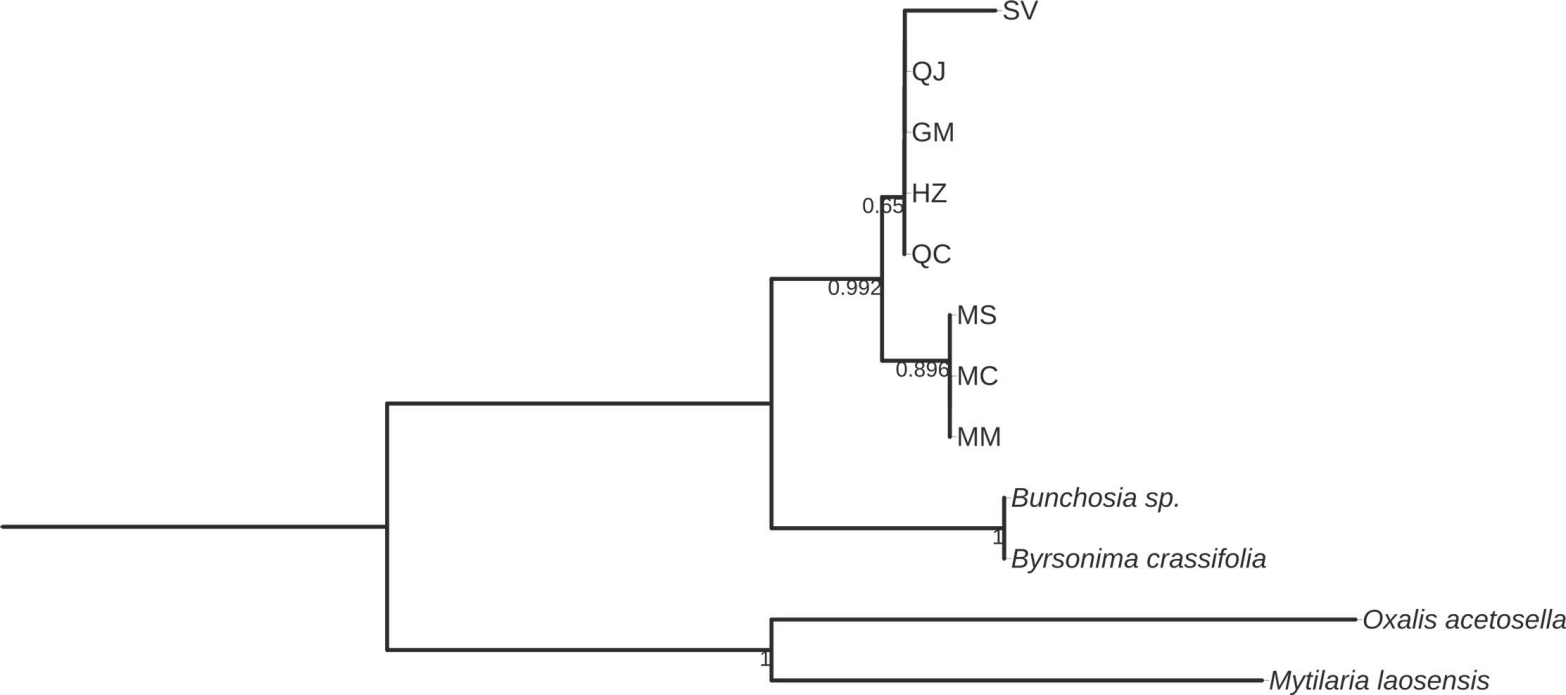
Bootstrap consensus tree generated by the Neighbor-joining method for *rpoC1* sequences obtained for *Galphimia* populations. Numbers below the branches are bootstrap values expressed as a percentage of 1000 replicates.

**Fig 5:**
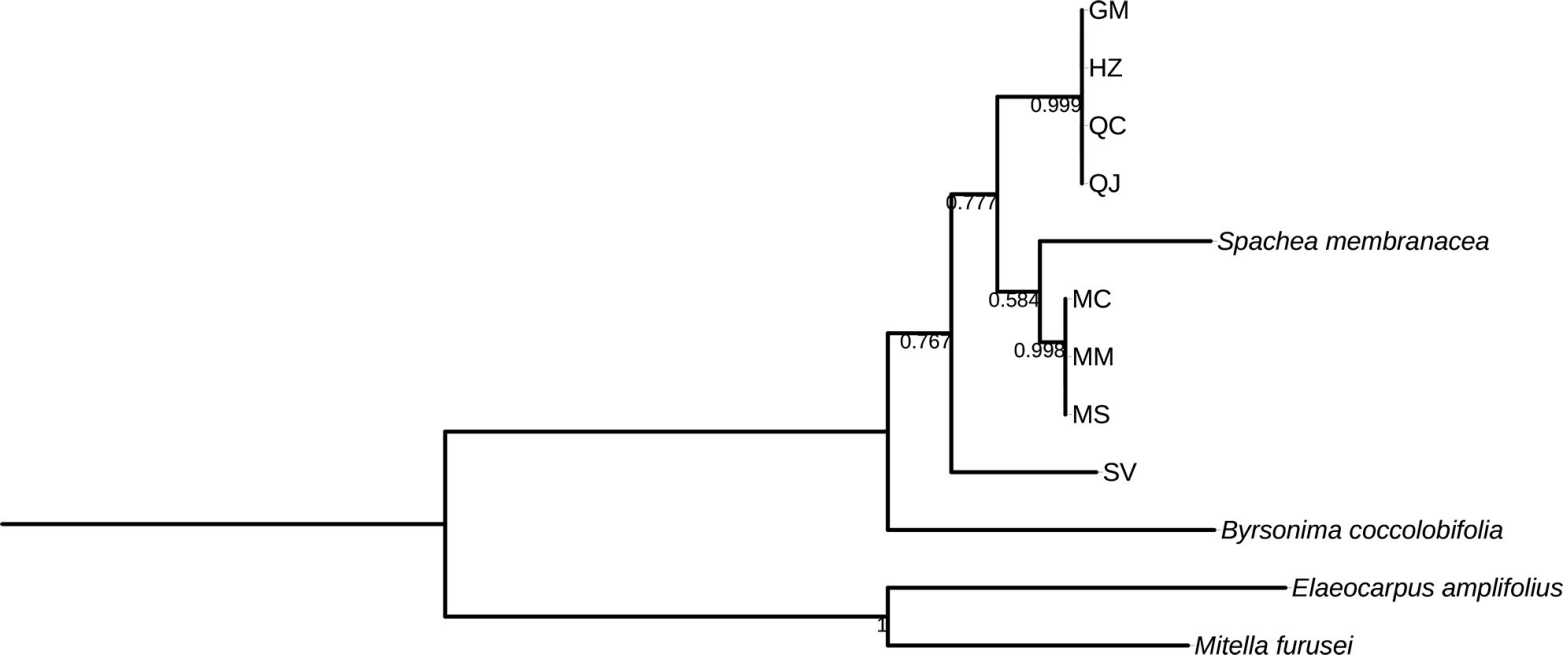
Bootstrap consensus tree generated by the Neighbor-joining method for *psbA-trnH* sequences obtained for *Galphimia* populations. Numbers below the branches are bootstrap values expressed as a percentage of 1000 replicates.

**Fig 6:**
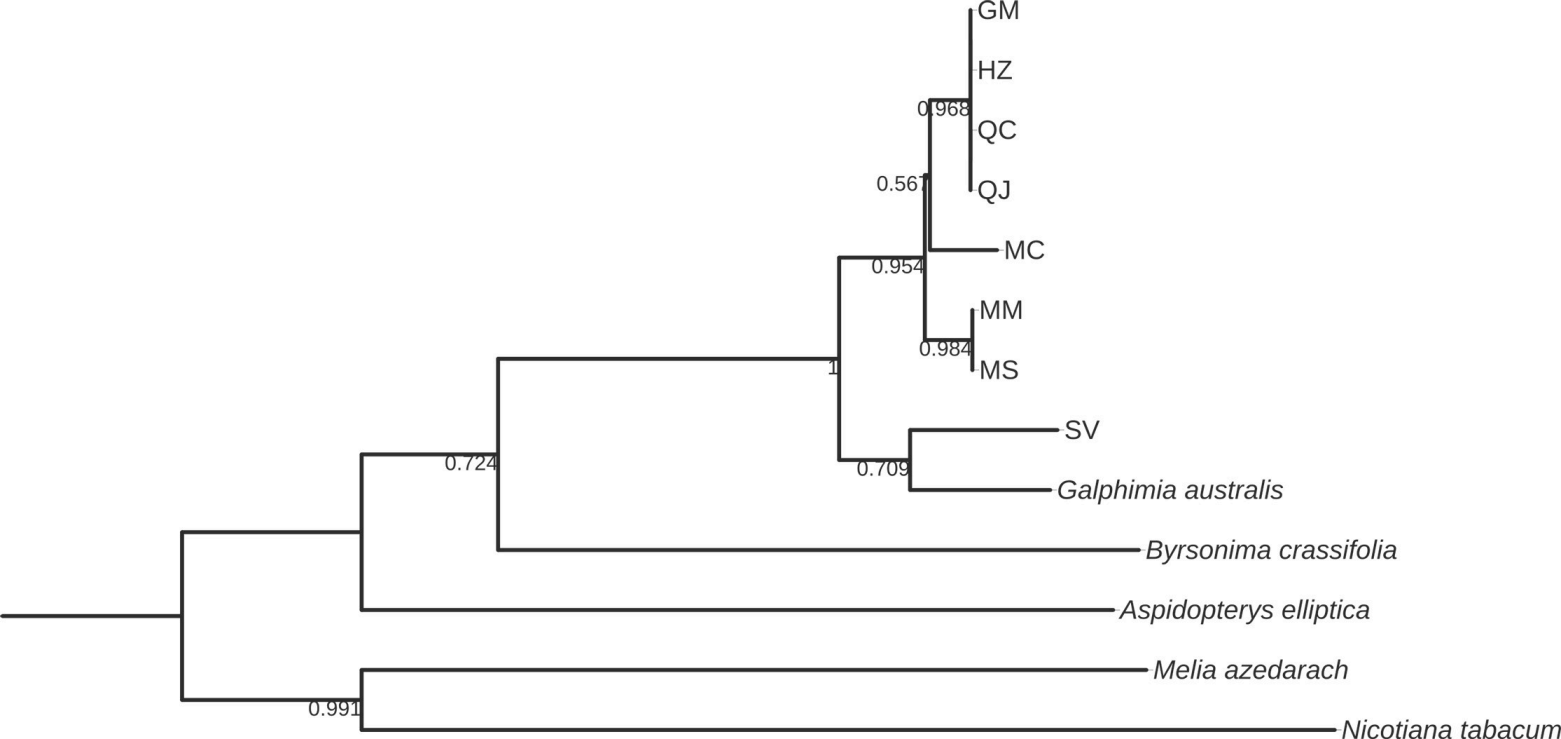
Bootstrap consensus tree generated by the Neighbor-joining method for *ITS1* sequences obtained for *Galphimia* populations. Numbers below the branches are bootstrap values expressed as a percentage of 1000 replicates.

**Fig 7:**
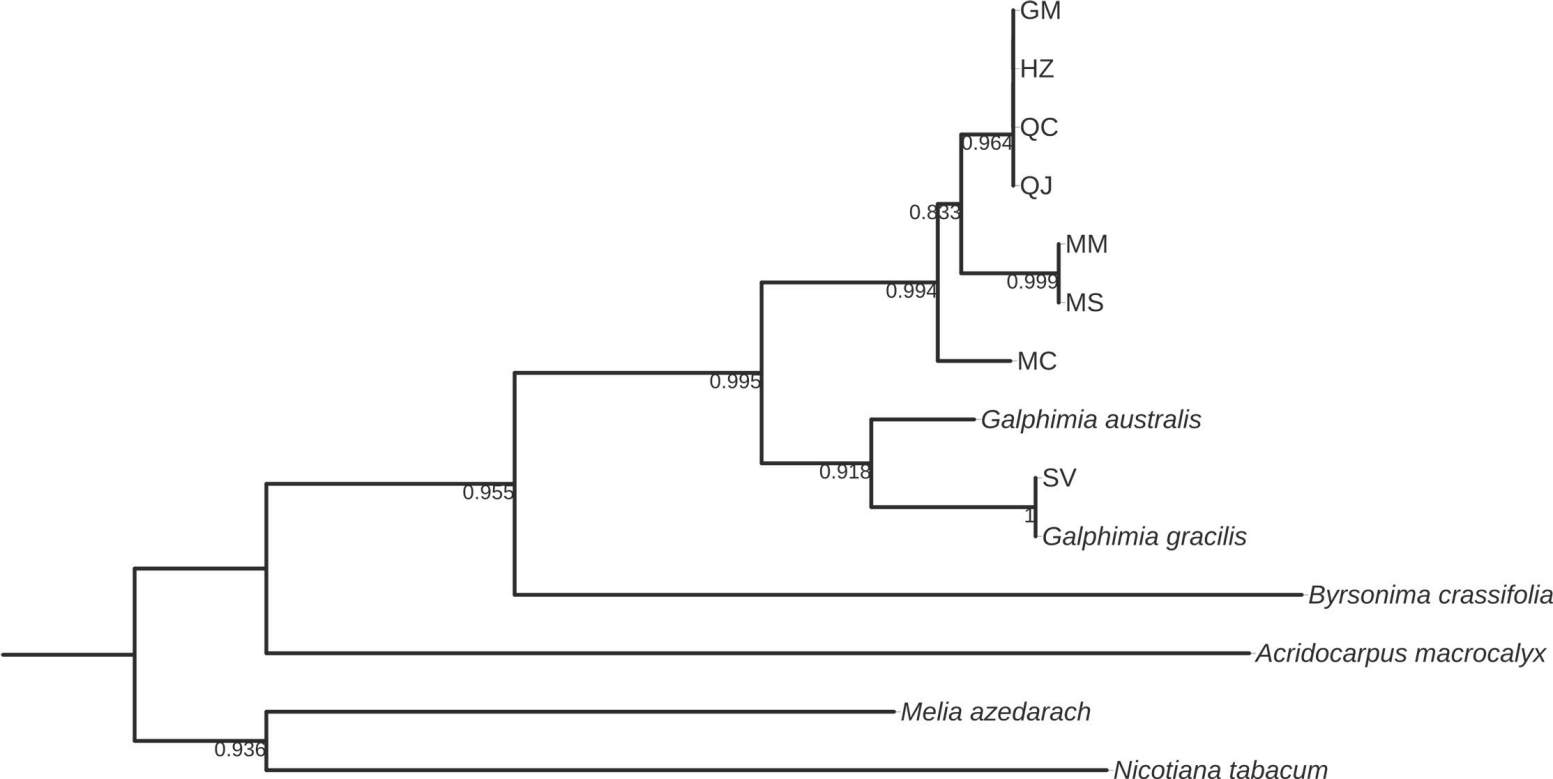
Bootstrap consensus tree generated by the Neighbor-joining method for *ITS2* sequences obtained for *Galphimia* populations. Numbers below the branches are bootstrap values expressed as a percentage of 1000 replicates.

**Fig 8:**
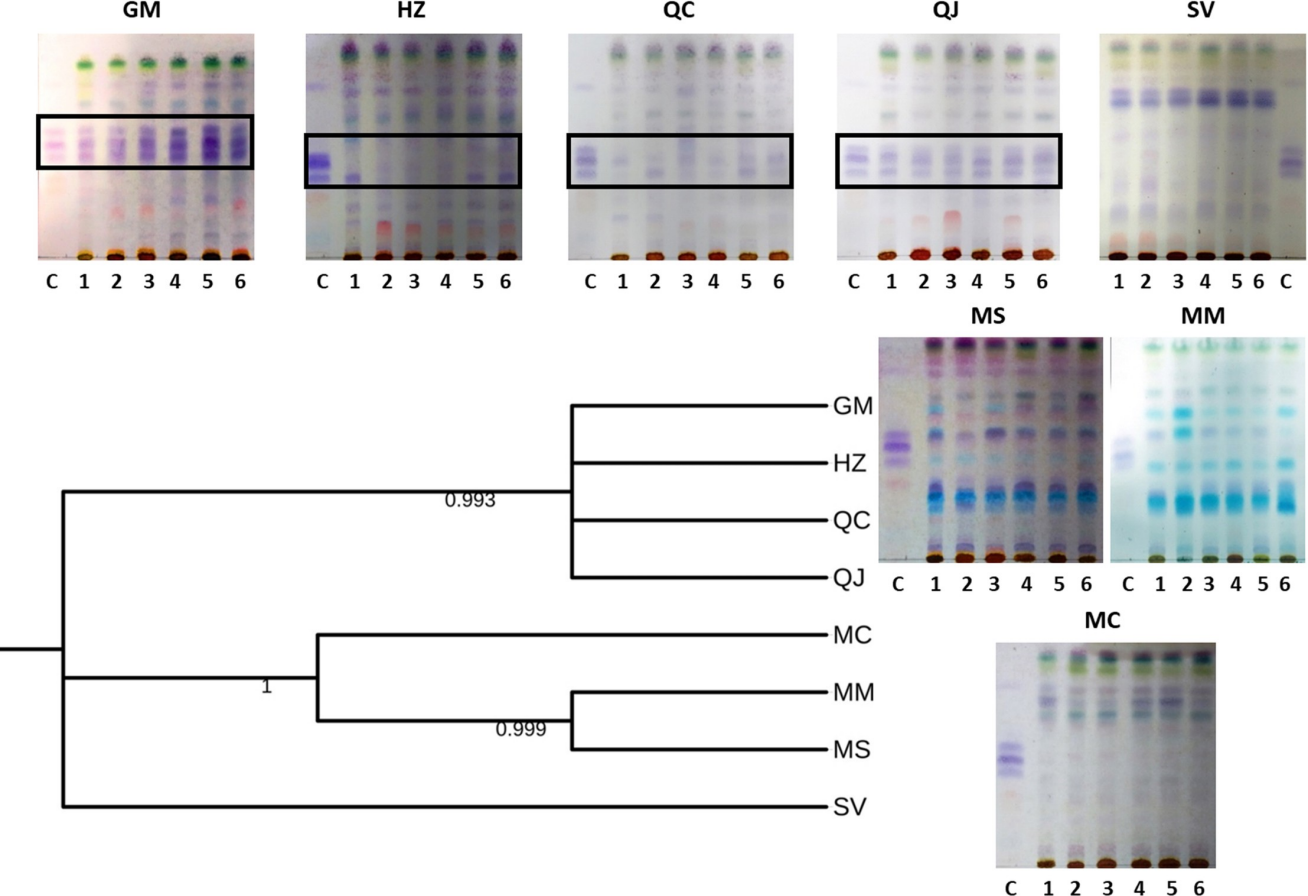
Comparison of Neighbor-joining tree for *Galphimia* populations analyzed in this study based on *matK*, *rbcL*, *rpoC1*, *psbA-trnH*, *ITS1*, and *ITS2* combined sequences and TLC profiles of methanolic extracts of individuals from these populations. The TLC mobile phase was CH_3_COOCH_2_CH_3_-CHCl_3_ (2:1). The individuals of each population are numbered (1–6). The control (C) is a fraction of galphimines that was obtained from individuals of GM. The presence of galphimines is indicated with a black frame.

The alignments for all six markers were developed, and three bootstrap consensus trees were constructed for each marker by means of NJ, ML, and MP methods. All phylogenetic trees showed strongly supported clades with high bootstrap values. Additionally, the bootstrap consensus trees obtained by the three methods were highly similar for each of the six DNA barcodes studied. The bootstrap values observed by ML and MP (S1 Appendix) were also highly similar with the trees of NJ analysis (Figs 2–8). The ML and MP methods support the results obtained by the NJ method.

Three and two major clades were formed for *matK*, *rbcL*, and *rpoC1* (Figs 2–4), and for *psbA-trnH*, *ITS1* and *ITS2* (Figs 5–7) genes, respectively. In the case of *matK*, four well-supported sister clades enabled us to separate the populations of GM, HZ, QC, and QJ from the populations of MC, MM, and MS. Furthermore, for the *matK* gene, the population of SV separated independently from the rest of the populations analyzed in this study (Fig 2). Similarly, for *rpoC1*, four well-supported sister clades showed discrimination between the populations collected in GM, HZ, QC, and QJ from the populations of MC, MM, and MS, and once again, the population of SV was kept apart in a different group from the rest of the studied populations (Fig 4). In accordance with the previous results, *psbA-trnH* allowed the segregation of the populations of GM, HZ, QC, and QJ from the populations of MC, MM, and MS, as well as the independent separation of the SV population (Fig 5). The *ITS1* and *ITS2* markers presented high rates of polymorphism, and once more, it was possible to discriminate among the populations of MC, MM, MS, and SV from the populations that came from GM, HZ, QC, and QJ (Figs 6 and 7). The phylogenetic analyses of *ITS1* and *ITS2* also showed that SV discriminated in an independent group (Figs 6 and 7). The *rbcL* gene did not show sufficient variation to differentiate among most of the studied populations but enabled us to discriminate one of them (SV) (Fig 3).

### Thin-layer chromatography analysis

The TLC profiles of *Galphimia* populations showed differences between them (Fig 8). Using this separation technique, it was possible to distinguish four principal groups: the galphimine-producing (marked with a black frame) populations (GM, HZ, QC and QJ) (group 1) and the nongalphimine-producing populations, which were separated in MM and MS (group 2), MC (group 3) and SV (group 4) (Fig 8). The bootstrap consensus tree based on *matK*, *rbcL*, *rpoC1*, *psbA-trnH*, *ITS1* and *ITS2* combined sequences presented four sister clades that corresponded with the four different groups observed in the TLC chemical profiles of the populations investigated in this study (Fig 8).

## Discussion

### DNA barcoding analysis

#### DNA barcode features and distance-based methods

The analysis of the genetic variability revealed that *rbcL* was the less polymorphic barcode, exhibiting no sites with gaps in the alignment and parsimony informative sites and exhibiting the lowest number of mutation and haplotypes. These results were similar to those previously obtained by our group [30] and to the findings of other studies developed in woody plant species from Brazil [37].

The values for the intraspecific distances for *matK* and *rpoC1* were low but enabled us to visualize a small differentiation between the populations that produce galphimines (GM, HZ, QC, and QJ) from those that do not produce these compounds (MC, MM, MS, and SV). These results are also consistent with the three haplotypes presented for *matK* and *rpoC1*. In addition, the *psbA-trnH* region showed three different haplotypes, which corresponded with the populations that produced galphimines (GM, HZ, QC, and QJ) and nonproducers that separated into two groups: one was integrated by the populations from Morelos (MC; MM and MS), and the other was composed only of the SV population.

Conversely, the *psbA-trnH* region presented high values of intraspecific distances that strongly suggest that the populations in the study belong to more than one species of the genus *Galphimia*. For the *ITS1* and *ITS2* intergenic regions, the results of the genetic variability analysis showed four haplotypes, and the high interspecific distances among the sequences of these markers suggest the presence of four different species of the genus *Galphimia* separated into four groups: the galphimine-producer populations [GM, HZ, QC, and QJ] and the nonproducers, which can be separated into three groups: [MC], [MM and MS] and [SV]. The results for *psbA-trnH*, *ITS1* and *ITS2* were expected because these intergenic regions have been reported as highly polymorphic barcodes in angiosperm [31,37–39], including medicinal species [18,32,52–54].

The lower values for the intraspecific distances displayed by the combination of all six DNA barcodes compared with the three analyzed intergenic regions are the result of small intraspecific distances of *matK*, *rbcL* and *rpoC1* markers. However, the results obtained for the combination of all six markers also support the presence of four different species of the genus.

#### Phylogenetic analysis

Numerous DNA sequences of *matK* and *rbcL* deposited in GenBank for the genus *Galphimia* were incorporated in this analysis with the objective of investigating the relationship with the samples described in this study. In the case of both *rpoC1* and *psbA-trnH*, we could not find any sequence for the genus *Galphimia*. For *ITS1* and *ITS2*, we found one and two sequences of the genus *Galphimia* deposited in the GenBank database, respectively. Moreover, other sequences that belong to species related and unrelated to *Galphimia* were included in the phylogenetic analysis to construct out groups for all six markers.

Most of the sequences of *matK* and *rbcL* for the genus *Galphimia* were reported previously [29], but some plants studied in that investigation did not receive an accession number and were not available in GenBank. The accession numbers in GenBank for *G*. *glauca* were reported by Tokuoka and Tobe [28] before the botanical classification for the genus *Galphimia* was published by Anderson [2]. The taxonomic classification as *G*. *glauca* related to these two sequences was made in the Kyoto Botanical Garden [28]. It is possible that this analysis was misinterpreted with other species of the genus because the population growing in Dr. Mora, Guanajuato (GM), was classified as *G*. *glauca* [2], but in this work and in a previous study developed by our group [30], it was demonstrated that the GM population is genetically different from *G*. *glauca* sequences reported formerly [28] for both *matK* and *rbcL* genes (Figs 2 and 3). The samples from SV grouped individually from the rest of the populations in study for *matK* and *rbcL* sequences (Figs 2 and 3). These samples were more closely related to *G*. *gracilis* for these two markers. In addition, SV was the population that presented the highest rate of polymorphisms among all of the populations in study. The results of the analysis of the *matK*, *rbcL* and *rpoC1* genes of this investigation corroborate our previous study in which *matK* and *rpoC1* were the markers that enabled us to discriminate among the galphimine producer populations and suggest the presence of three different species of the genus *Galphimia*.

In this work, we report the phylogenetic analyses of *psbA-trnH*, *ITS1* and *ITS2* sequences in *G*. *glauca* for the first time. These barcode sequences show a high level of DNA variation among the eight natural populations of *G*. *glauca* that we analyzed. The high level of variability observed for these three markers was expected because spacer DNA sequences, as noncoding DNA regions, show low selection pressure and exhibit a high level of mutation rates [55,56]. The phylogenetic trees that were obtained by NJ, ML, and MP based on *psbA-trnH*, *ITS1* and *ITS2* also allowed the discrimination of the galphimines producing populations (GM, HZ, QC, and QJ) from the rest of the populations, which do not produce galphimines (MC, MM, MS, and SV). With the use of *ITS1* and *ITS2* sequences, for the first time, it was possible to discriminate two groups from the populations growing in Morelos: group 1 is formed by the MC population, and group 2 comprises MM and MS populations (Figs 6 and 7). This result suggests that both MS and MM belong to the same species of the genus *Galphimia*. Although the *psbA-trnH* barcode was highly polymorphic, it did not enable the differentiation of the populations growing in the state of Morelos.

DNA barcoding is a tool that allowed us to distinguish among the specimens of the genus *Galphimia*, as in the previous work of our group [30]. The use of the DNA barcodes *ITS1* and *ITS2* was useful to show a genetic variability that had not been identified previously.

### Thin-layer chromatography analysis

TLC is a simple, reproducible and easy technique that represents a useful strategy to analyze chemical profiles. We used TLC to correlate the molecular results with the chemical profiles that were obtained in this study. In previous studies, it has been demonstrated that metabolic profiling is a reliable approach to study the chemical profiles of *Galphimia* populations [30], suggesting that galphimines could be a chemotaxonomic marker for *G*. *glauca* species. The variation in environmental factors is tightly related to the differential production of metabolites in plants. However, the genetic information variability is the primary cause of any phenotypic variation in organisms; consequently, the genetic variation that was demonstrated in this study could be the answer to the production of galphimines in four (GM, HZ, QC, and QJ) of the eight populations studied and correlates perfectly with the chemical profiles that were obtained by TLC (Fig 8). Potentially, these differences are related to the different genetic information among the populations described in this study. Similar results were found in our previous research [30].

The TLC profiles enabled us to differentiate among the populations of Morelos, MM and MS in contrast with MC. The first two populations presented a group of blue-colored compounds, revealed by vanillin/H_2_SO_4_, that were absent in the samples from the MC population. The differences in the production of metabolites of the populations that we studied could be related to the diverse ecological regions from which they originated, but the molecular analysis also demonstrated that these differences could be due to different genetic information between these populations. Genetic and environmental factors are closely related, and the interaction between them modulates the response as a particular phenotype of all living organisms.

### Integration of phylogenetic and thin-layer chromatography analysis

The bootstrap consensus tree based on *matK*, *rbcL*, *rpoC1*, *psbA-trnH*, *ITS1* and *ITS2* combined sequences was compared with the TLC chemical profiles of the populations described in this study studied (Fig 8). The chemical profiles obtained by TLC were uniform, stable and highly reproducible. DNA barcode analysis was well supported by TLC profiles of these plant samples (Fig 8). Thus, TLC was effective in discriminating among populations that produce galphimines, populations with blue-colored compounds, and those that do not produce these two groups of metabolites. The different concentrations of the same metabolites among individuals of the same population are a consequence of each individual’s ability to respond differently to environmental changes. The phylogenetic analysis and the TLC chemical profiles evidently matched. The genotype analysis in the phylogenetic tree presented four sister clades that corresponded with four different phenotypes in the TLC chemical profiles. These results were similar to those obtained in the previous investigation performed by our group [30].

Phylogenetic analysis and TLC chemical profiles were convenient tools to establish a strong relationship between the genotype and phenotype of the populations in study. The phylogenetic analysis of all six DNA barcodes analyzed in this study suggests that the populations in this study belong to at least three different species of the genus *Galphimia*. This analysis would be complemented with an ongoing morphological investigation, as well as cytogenetic studies, to establish a powerful and integrative methodology for quality control purposes that allow the preparation of effective herbal medicines from plants of this genus.

## Supporting information

S1 Appendix Phylogenetic trees generated by maximum likelihood and maximum parsimony methods. (PDF)

S2 Appendix Herbarium specimens of the natural population in study botanically classified as *Galphimia glauca*. (PDF)

S1 Spreadsheet *matK* sequence alignment. (XLS)

S2 Spreadsheet *rbcL* sequence alignment. (XLS)

S3 Spreadsheet *rpoC1* sequence alignment. (XLS)

S4 Spreadsheet *psbA-trnH* sequence alignment. (XLS)

S5 Spreadsheet *ITS1* sequence alignment. (XLS)

S6 Spreadsheet *ITS2* sequence alignment. (XLS)
