# Supplementary material for: DNA barcoding and TLC as tools to properly identify natural populations of the Mexican medicinal species Galphimia glauca Cav
Source: PLoS One. 2019 May 28;14(5):e0217313. doi: 10.1371/journal.pone.0217313 (PMC6538163; doi:10.1371/journal.pone.0217313)
Supplement: S1 Appendix — (PDF) [file pone.0217313.s001.pdf]

S1 Appendix

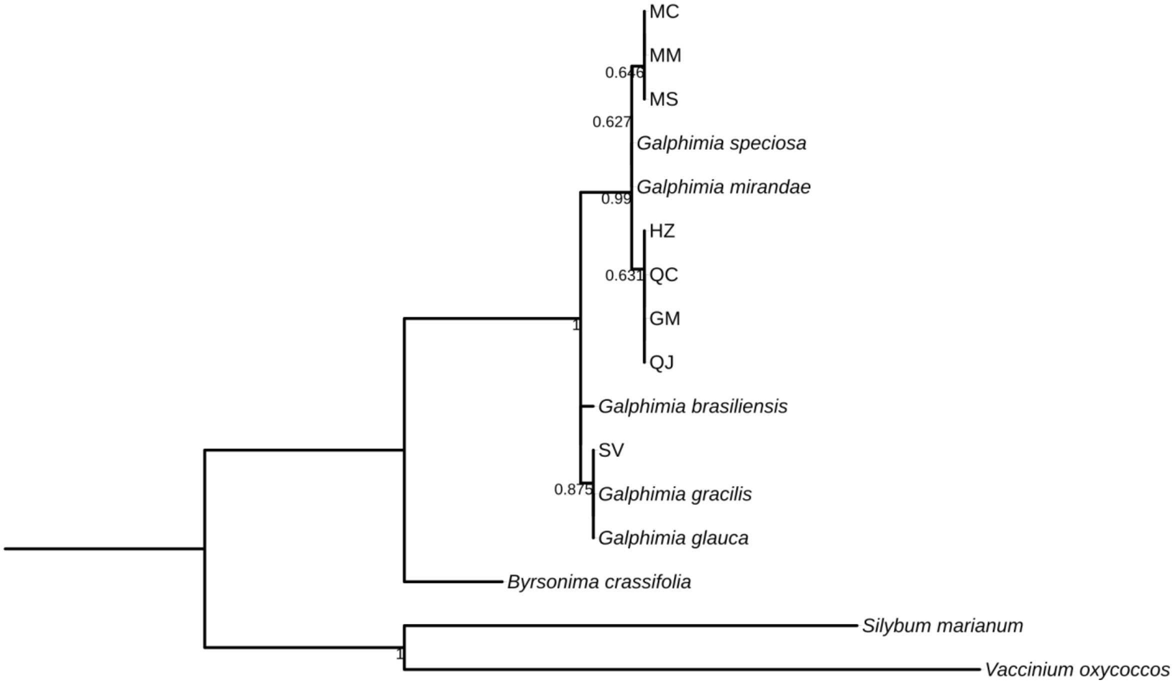

S1 Fig A. Bootstrap consensus tree generated by the Maximum Likelihood method for the *matK* sequences obtained for *Galphimia* populations here studied. Numbers below the branches are bootstrap values expressed as percentage of 1000 replicates.

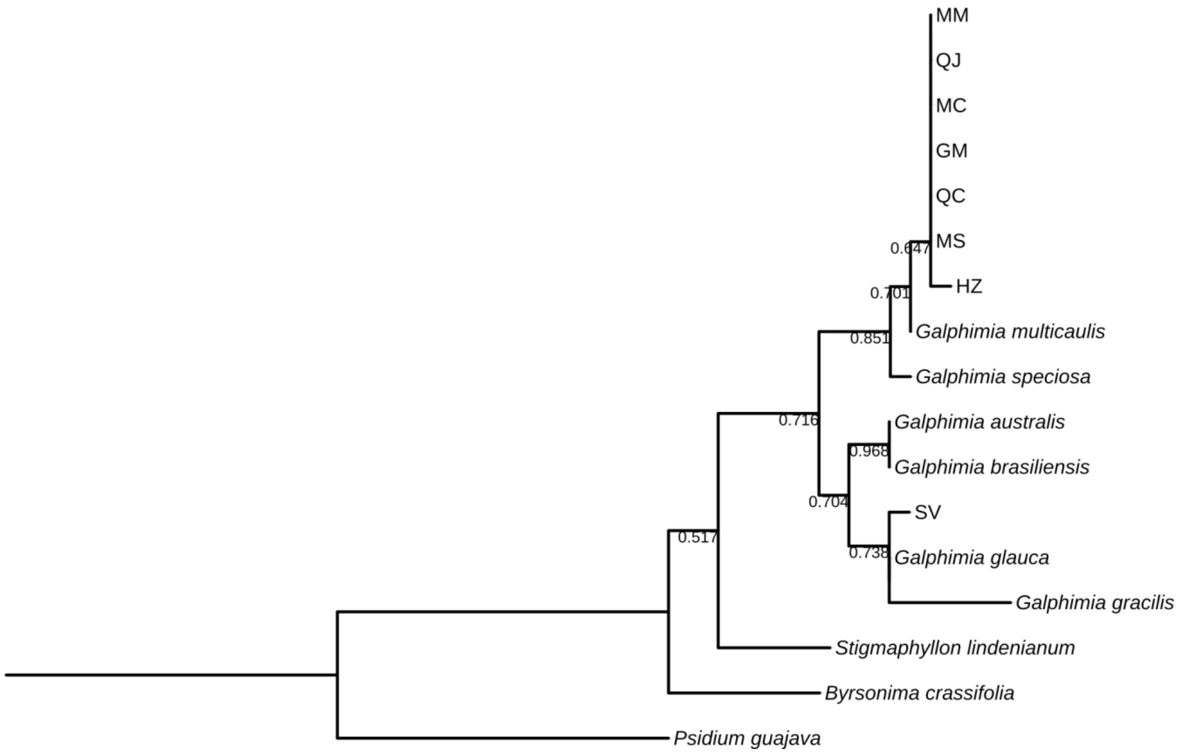

S1 Fig B. Bootstrap consensus tree generated by the Maximum Likelihood method for the *rbcL* sequences obtained for *Galphimia* populations here studied. Numbers below the branches are bootstrap values expressed as percentage of 1000 replicates.

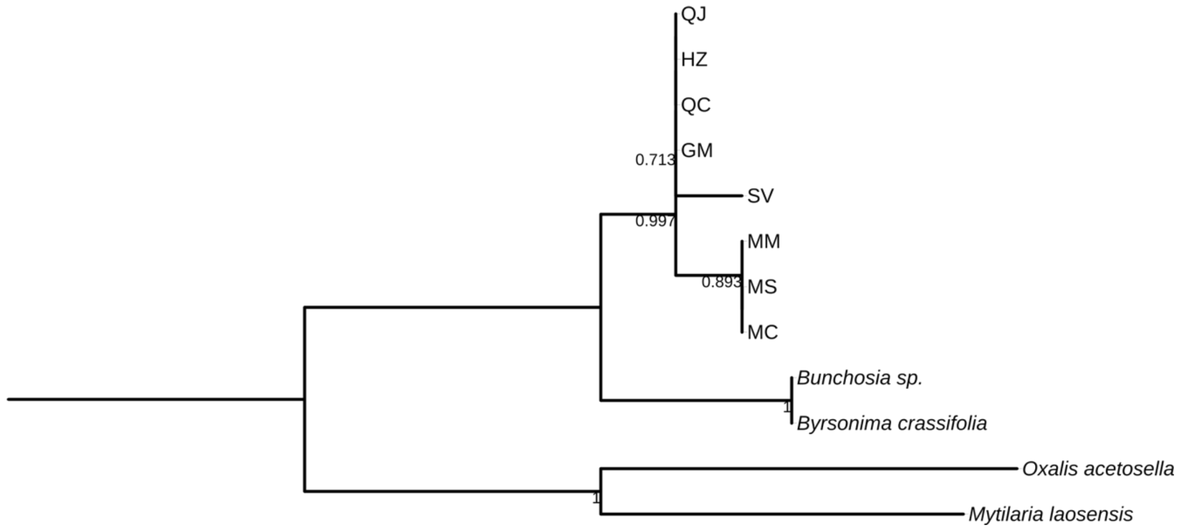

S1 Fig C. Bootstrap consensus tree generated by the Maximum Likelihood method for the *rpoC1* sequences obtained for *Galphimia* populations here studied. Numbers below the branches are bootstrap values expressed as percentage of 1000 replicates.

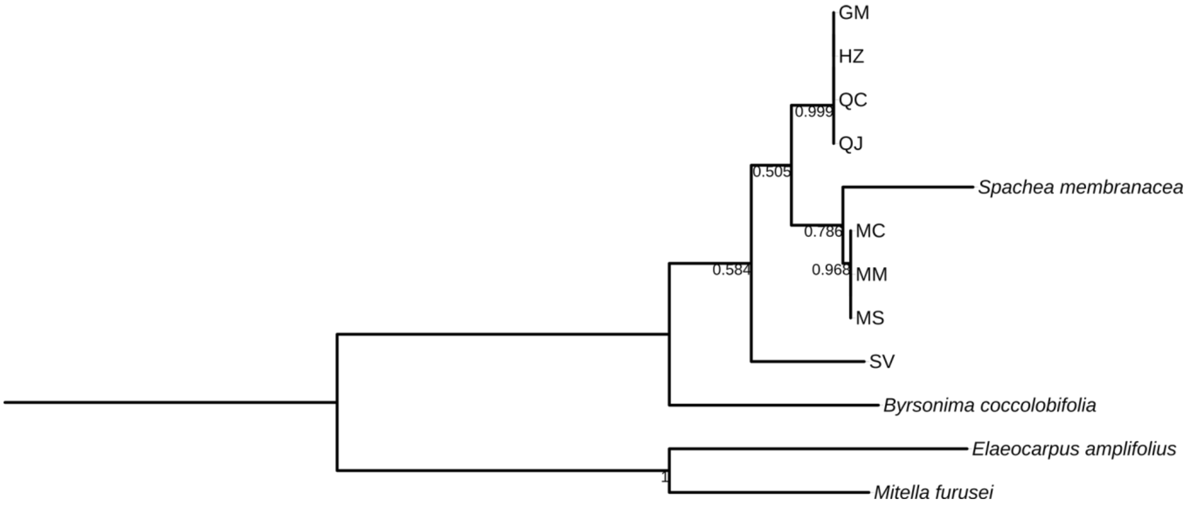

S1 Fig D. Bootstrap consensus tree generated by the Maximum Likelihood method for the *psbA-trnH* sequences obtained for *Galphimia* populations here studied. Numbers below the branches are bootstrap values expressed as percentage of 1000 replicates.

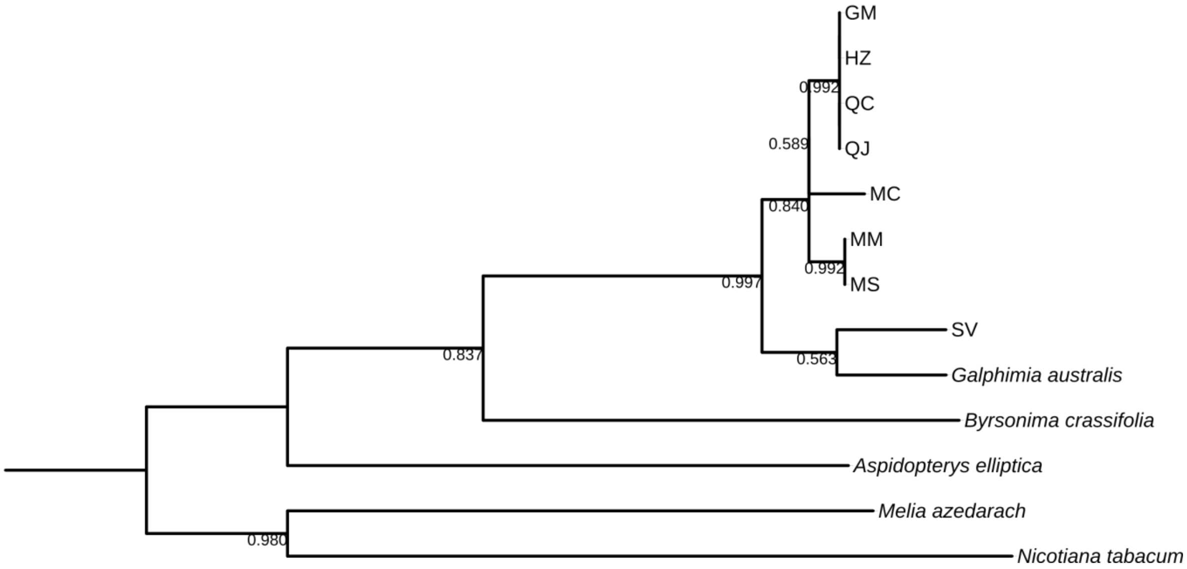

S1 Fig E. Bootstrap consensus tree generated by the Maximum Likelihood method for the *ITS1* sequences obtained for *Galphimia* populations here studied. Numbers below the branches are bootstrap values expressed as percentage of 1000 replicates.

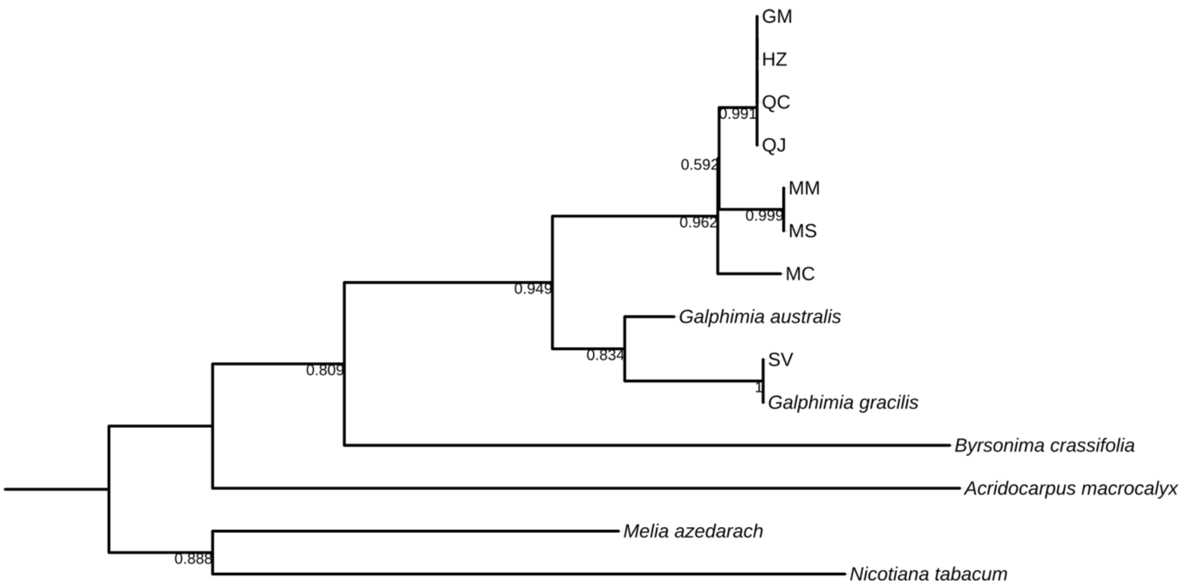

S1 Fig F. Bootstrap consensus tree generated by the Maximum Likelihood method for the *ITS2* sequences obtained for *Galphimia* populations here studied. Numbers below the branches are bootstrap values expressed as percentage of 1000 replicates.

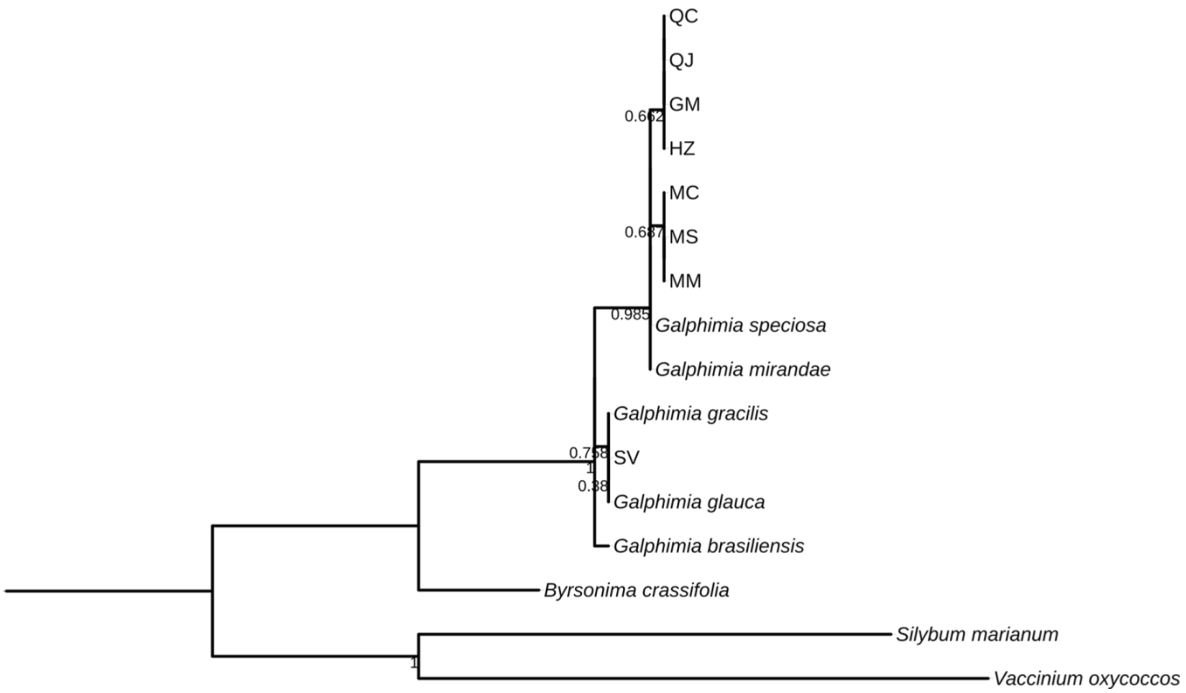

S1 Fig G. Bootstrap consensus tree generated by the Maximum Parsimony method for the *matK* sequences obtained for *Galphimia* populations here studied. Numbers below the branches are bootstrap values expressed as percentage of 1000 replicates.

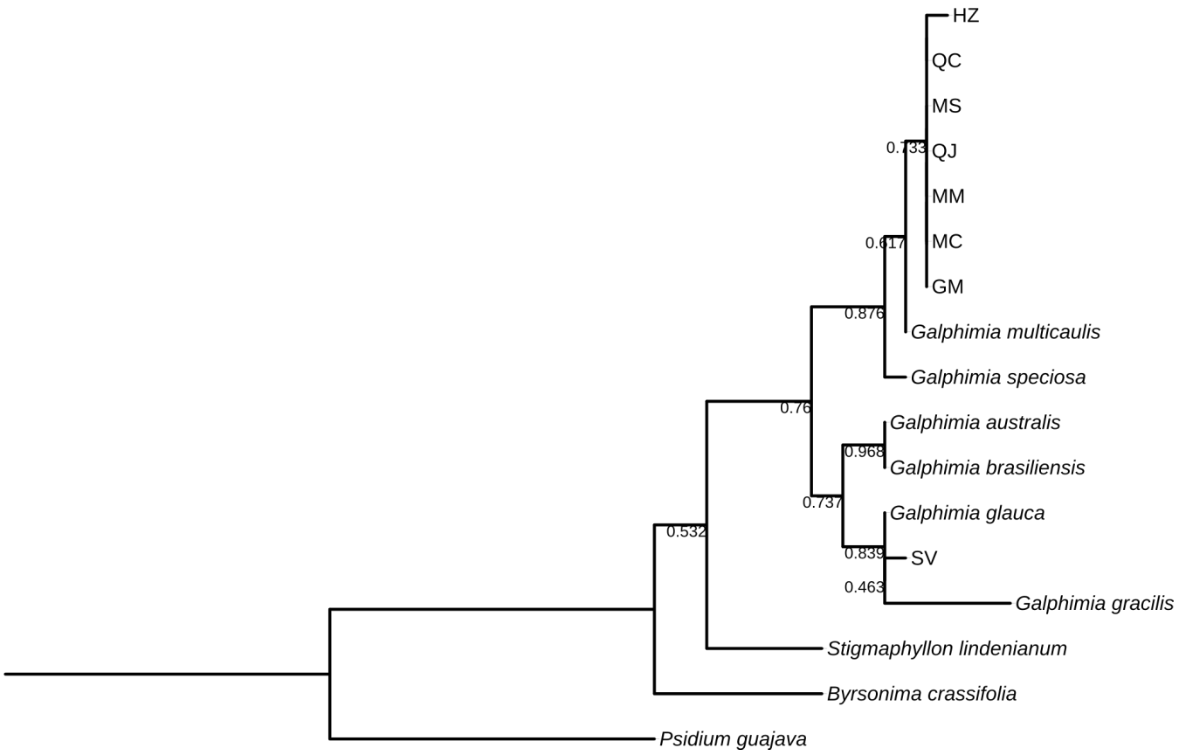

S1 Fig H. Bootstrap consensus tree generated by the Maximum Parsimony method for the *rbcL* sequences obtained for *Galphimia* populations here studied. Numbers below the branches are bootstrap values expressed as percentage of 1000 replicates.

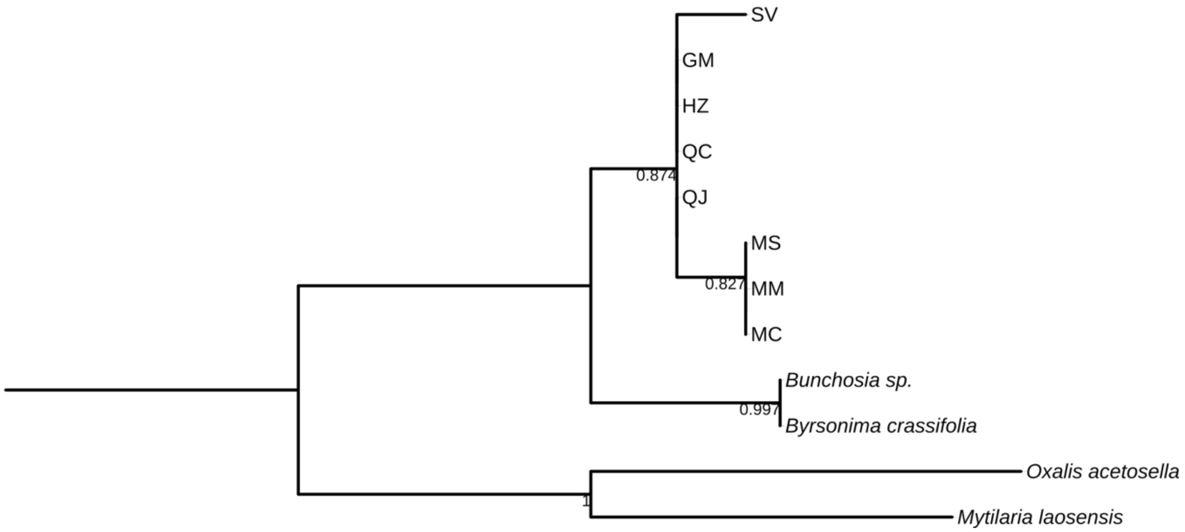

S1 Fig I. Bootstrap consensus tree generated by the Maximum Parsimony method for the *rpoC1* sequences obtained for *Galphimia* populations here studied. Numbers below the branches are bootstrap values expressed as percentage of 1000 replicates.

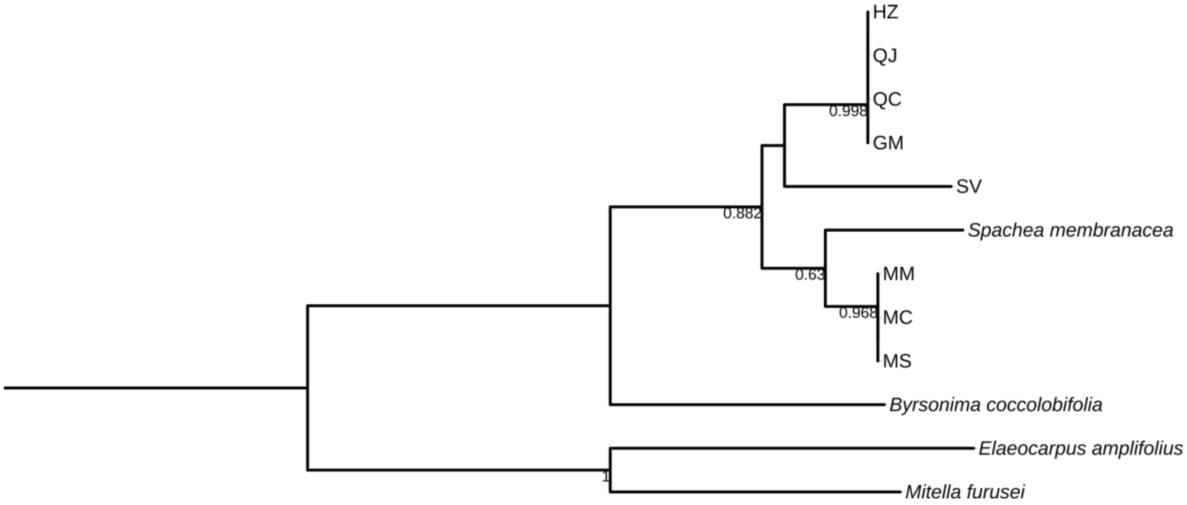

S1 Fig J. Bootstrap consensus tree generated by the Maximum Parsimony method for the *psbA-trnH* sequences obtained for *Galphimia* populations here studied. Numbers below the branches are bootstrap values expressed as percentage of 1000 replicates.

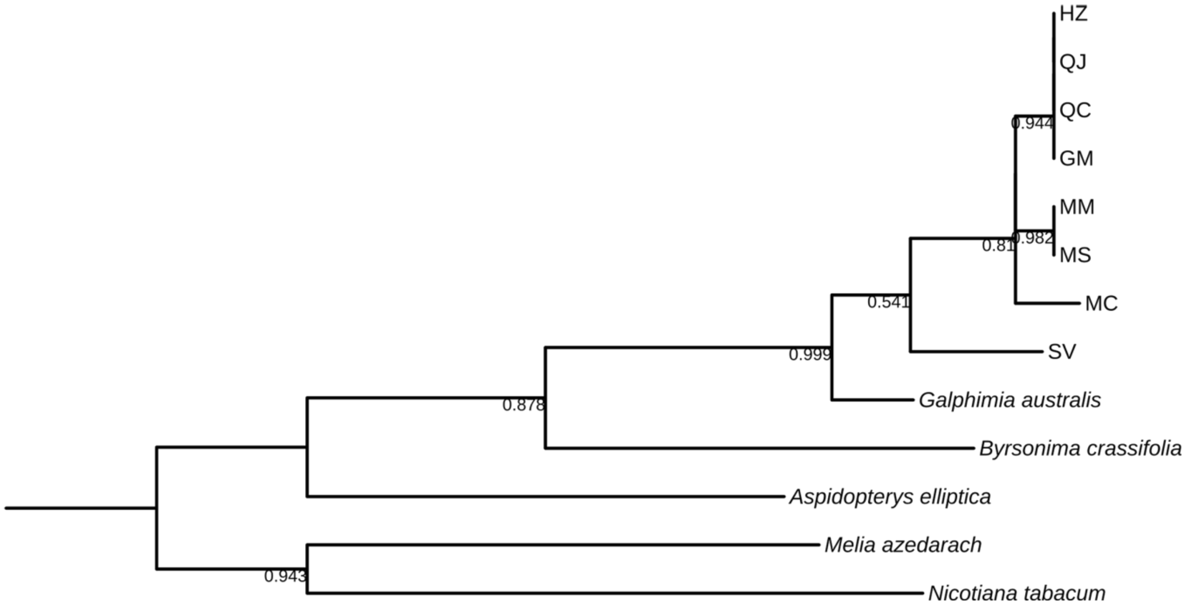

S1 Fig K. Bootstrap consensus tree generated by the Maximum Parsimony method for the *ITS1* sequences obtained for *Galphimia* populations here studied. Numbers below the branches are bootstrap values expressed as percentage of 1000 replicates.

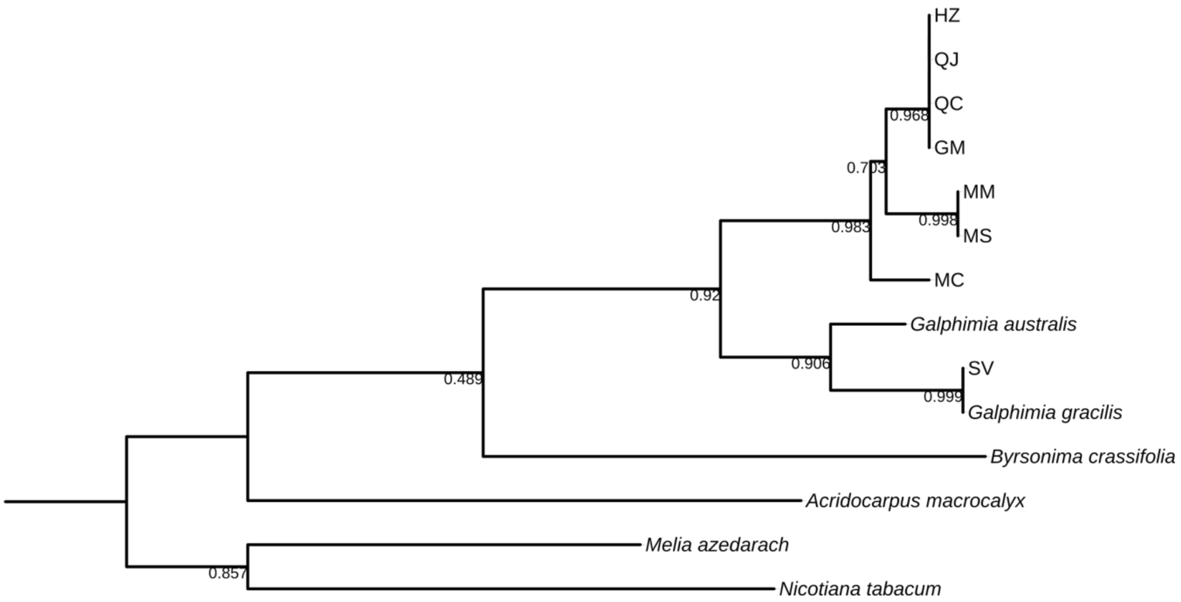

S1 Fig L. Bootstrap consensus tree generated by the Maximum Parsimony method for the *ITS2* sequences obtained for *Galphimia* populations here studied. Numbers below the branches are bootstrap values expressed as percentage of 1000 replicates.
