# Supplementary material for: DNA barcoding and TLC as tools to properly identify natural populations of the Mexican medicinal species Galphimia glauca Cav
Source: PLoS One. 2019 May 28;14(5):e0217313. doi: 10.1371/journal.pone.0217313 (PMC6538163; doi:10.1371/journal.pone.0217313)
Supplement: S2 Appendix — (PDF) [file pone.0217313.s002.pdf]

S2 Appendix

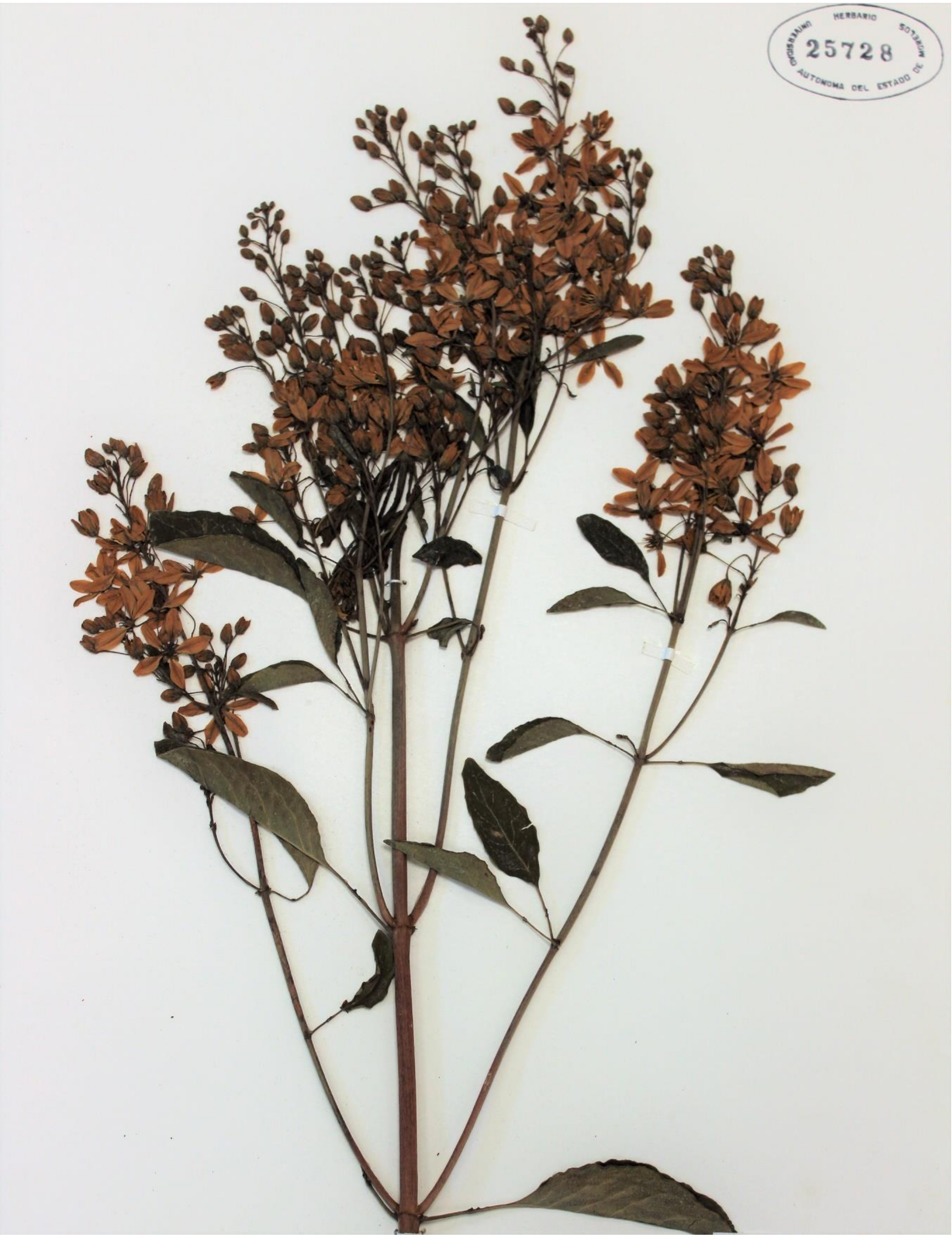

S2 Fig A. *Galphimia glauca* specimen from Dr. Mora, Guanajuato. The botanical classification was made at the HUMO Herbarium.

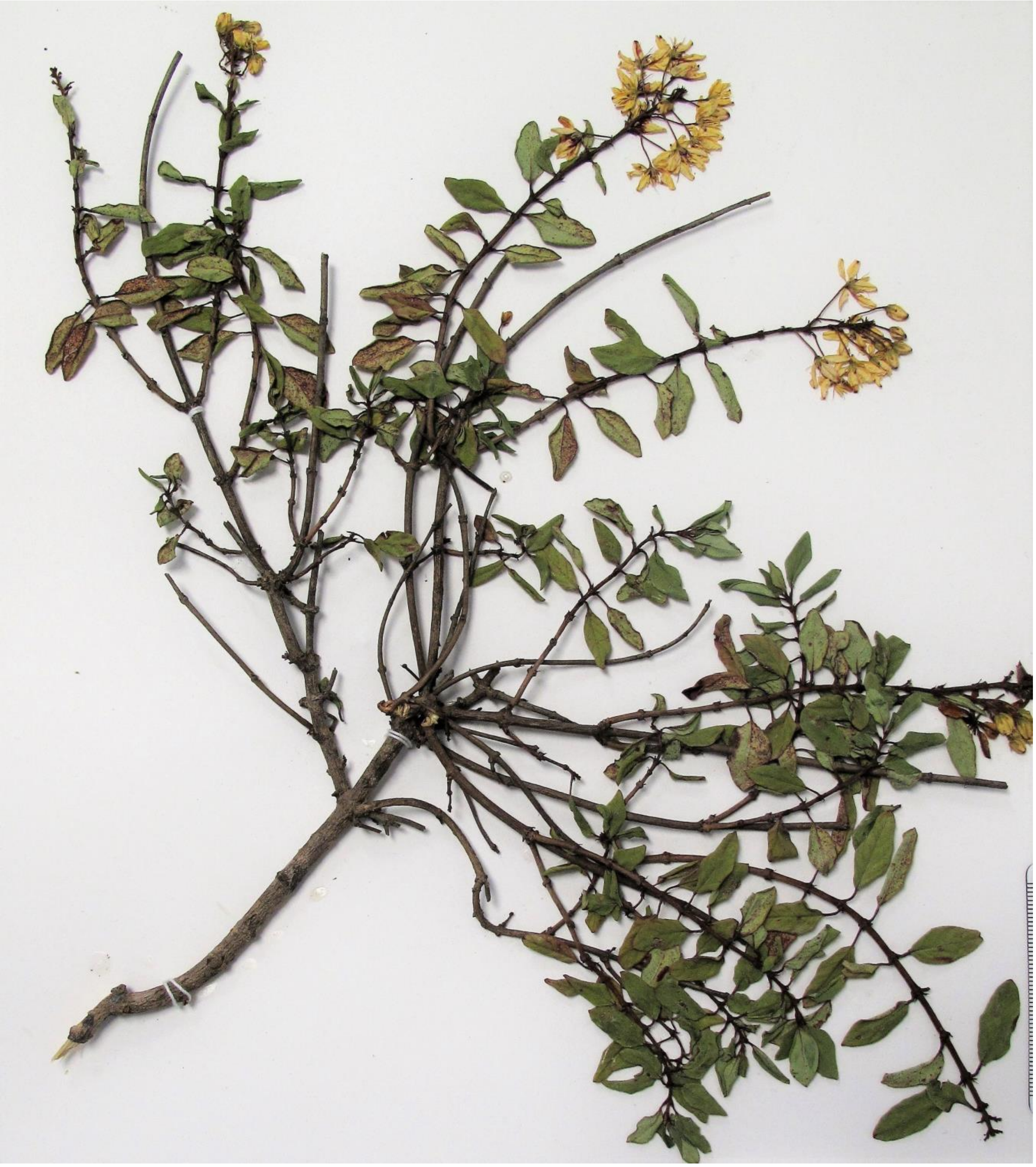

S2 Fig B. *Galphimia glauca* specimen from Zimapán, Hidalgo. The botanical classification was made at the HGOM Herbarium.

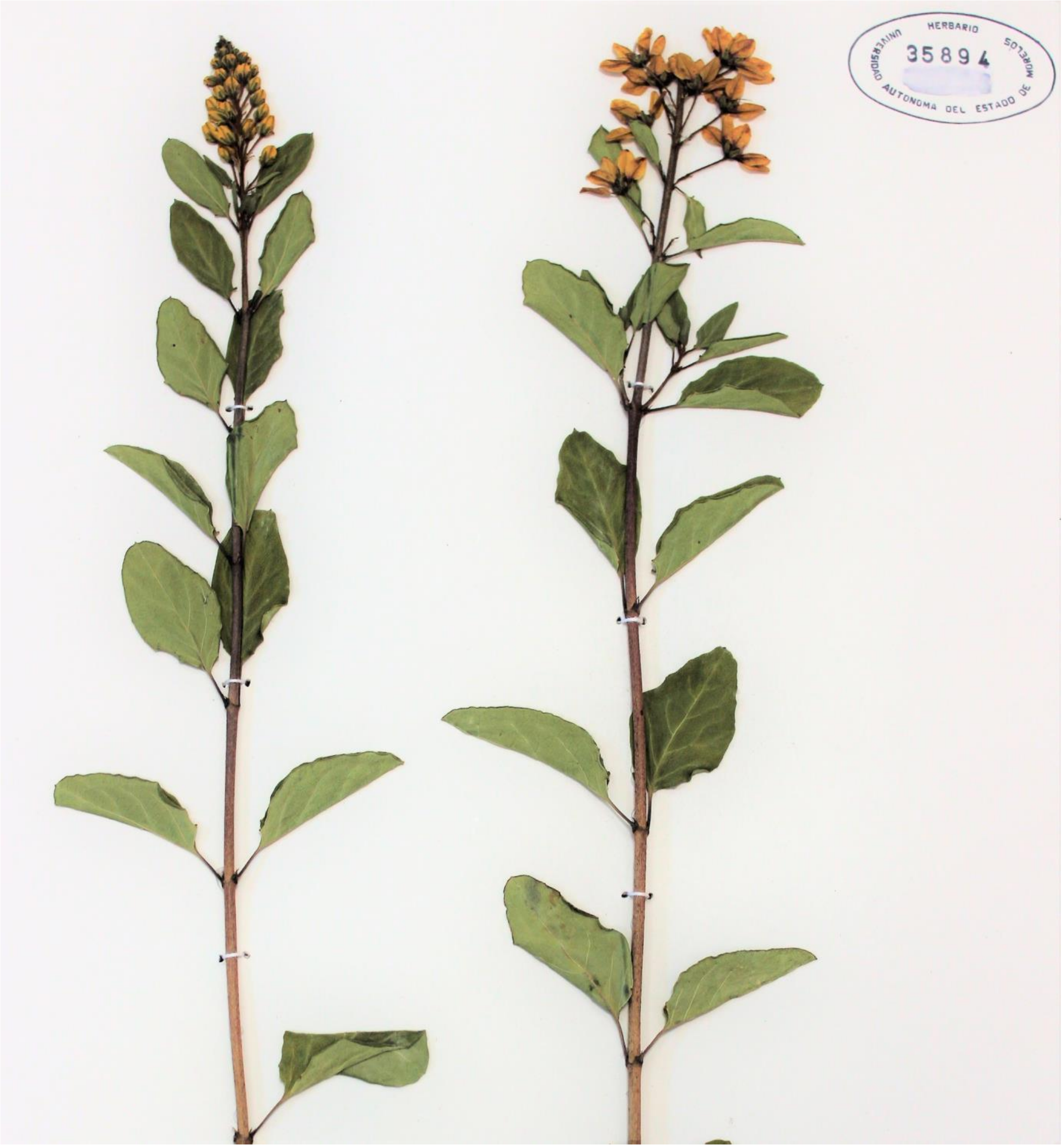

S2 Fig C. *Galphimia glauca* specimen from Cadereyta, Querétaro. The botanical classification was made at the HUMO Herbarium.

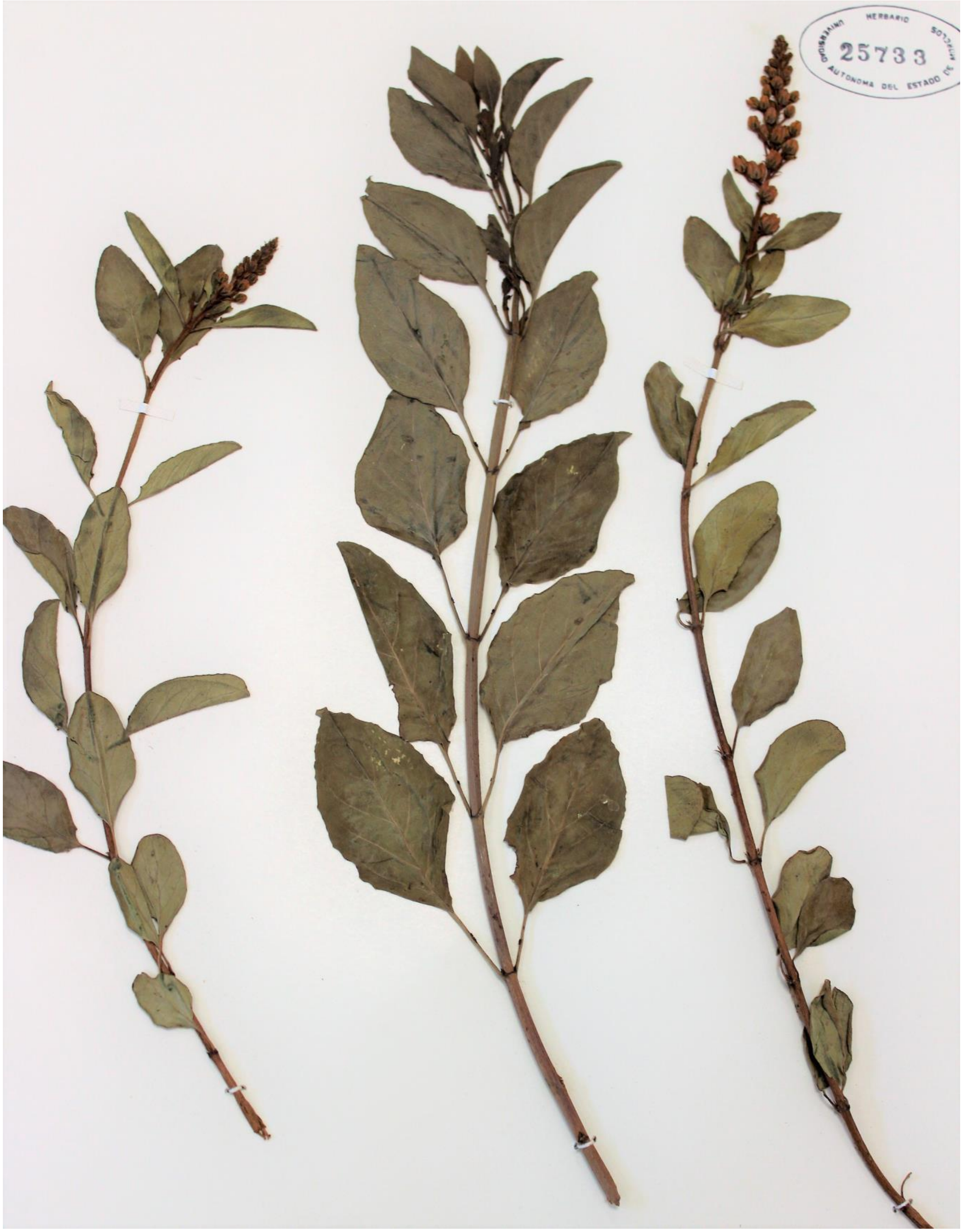

S2 Fig D. *Galphimia glauca* specimen from Jalpan de Serra, Querétaro. The botanical classification was made at the HUMO Herbarium.

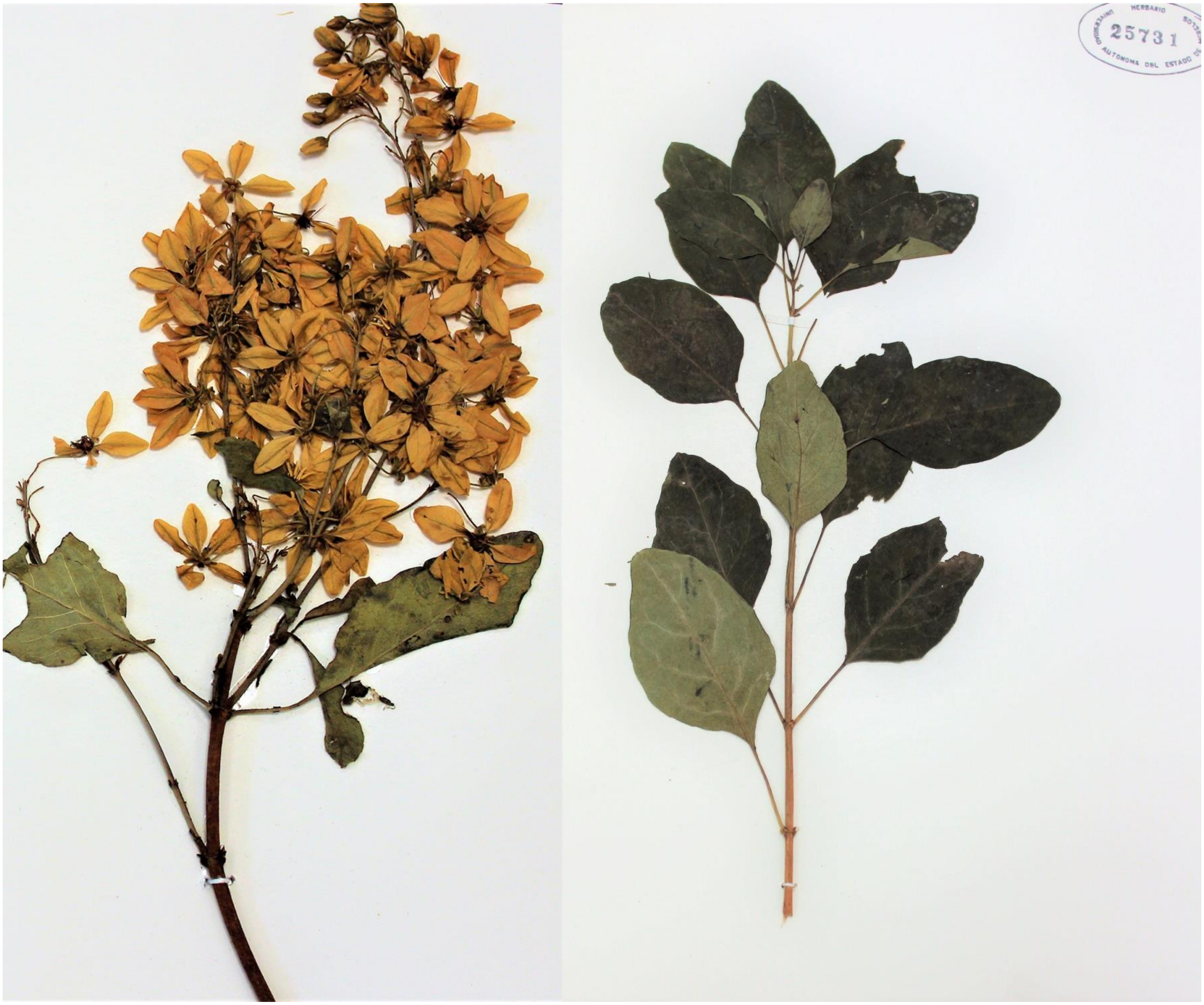

S2 Fig E. *Galphimia glauca* specimens from Cuernavaca, Morelos. The botanical classification was made at the HUMO Herbarium.

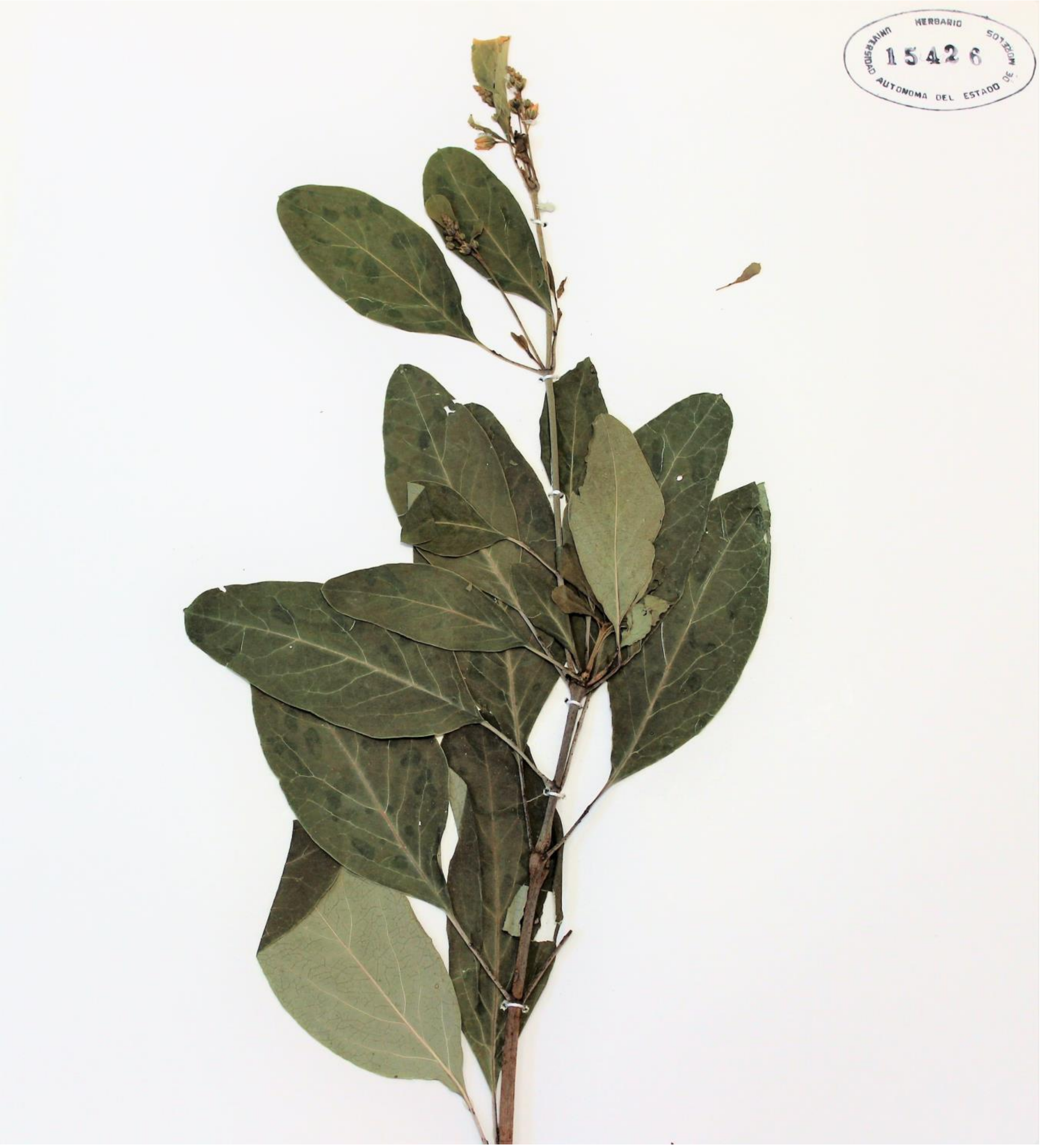

S2 Fig F. *Galphimia glauca* specimen from Miacatlán, Morelos. The botanical classification was made at the HUMO Herbarium.

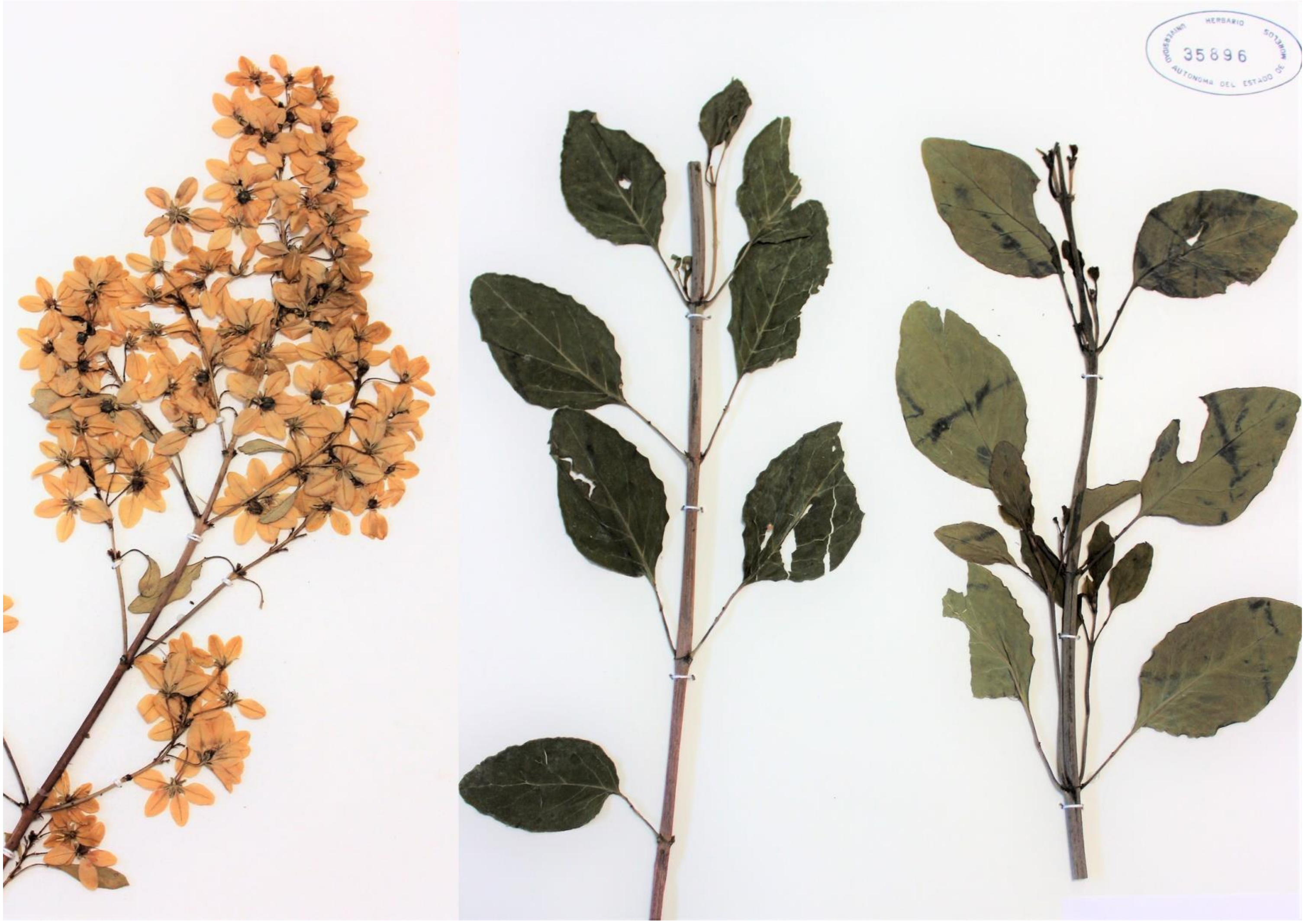

S2 Fig G *Galphimia glauca* specimens from Santa Catarina, Morelos. The botanical classification was made at the HUMO Herbarium.

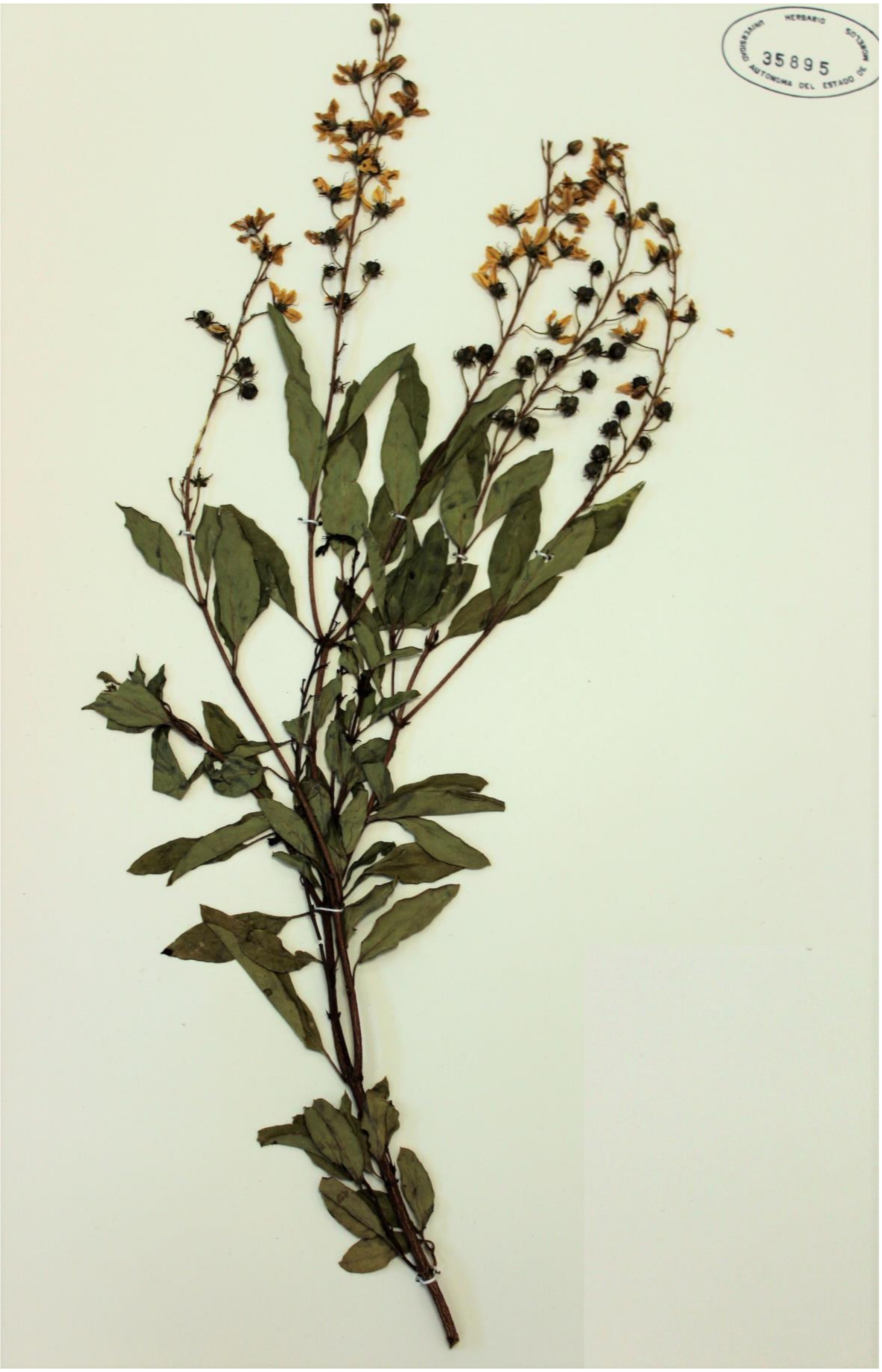

S2 Fig H. *Galphimia glauca* specimen from Ciudad Valles, San Luis Potosí. The botanical classification was made at the HUMO Herbarium.
